# Supplementary figures and images for: BRD4 modulates antimicrobial defense via non-canonical NRF2 activation in macrophages to confer protection against sepsis
Source: PLoS Pathog. 2026 Apr 30;22(4):e1014192. doi: 10.1371/journal.ppat.1014192 (PMC13155688; doi:10.1371/journal.ppat.1014192)

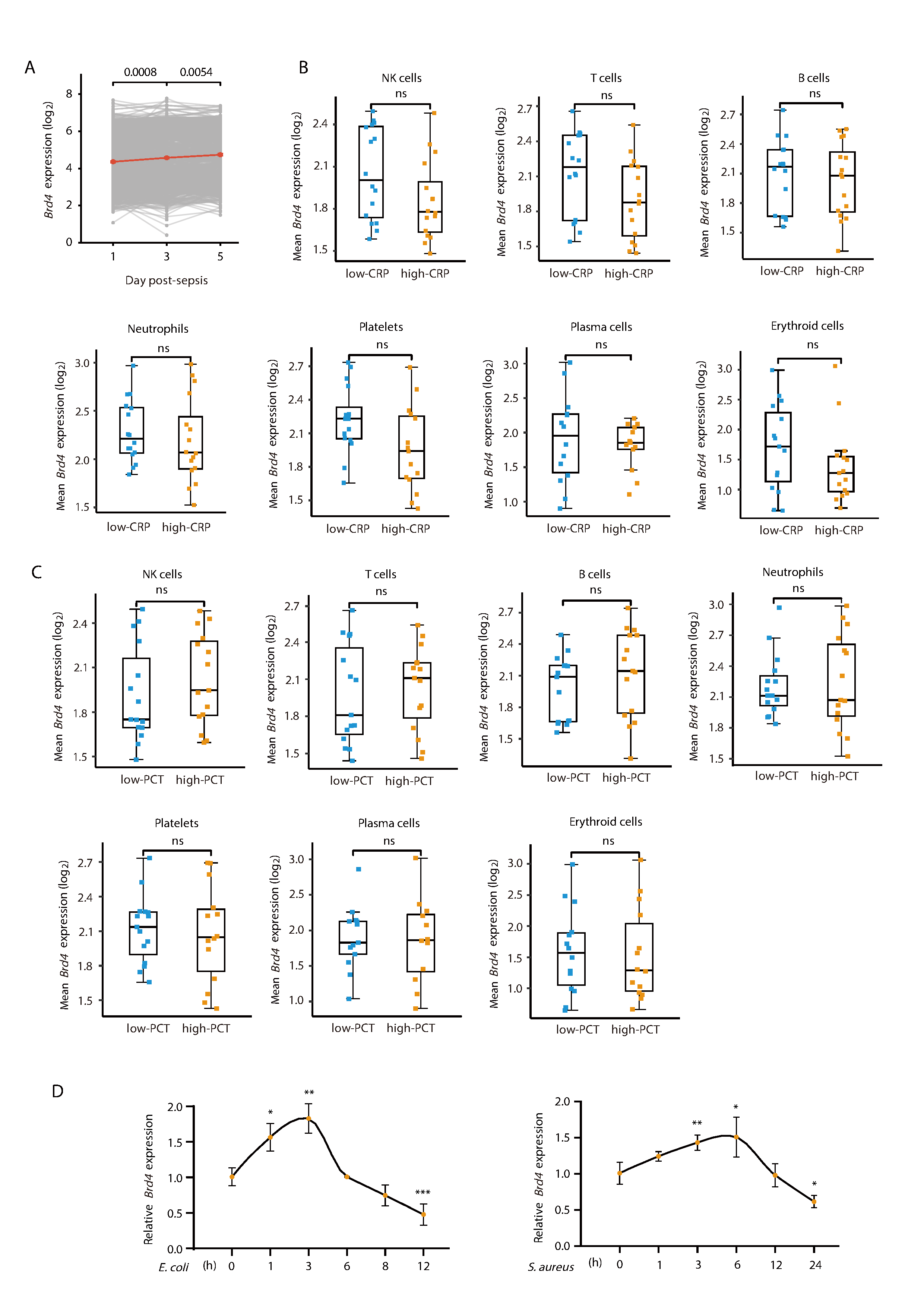

Supplement: S1 Fig — (A) Linear mixed-effects model analysis of BRD4 levels in septic patients across Days 1, 3, and 5. (B) Comparison of BRD4 mRNA levels in various immune cells between low-CRP and high-CRP septic patients. Data sources: CMAISE. (C) Comparison of BRD4 mRNA levels in various immune cells between low-PCT and high- PCT septic patients. Data sources: CMAISE. (D) Quantification of Brd4 mRNA levels in BMDMs at different time points following induction with E. coli (left) or S. aureus (right) by qRT-PCR. (TIF) [file ppat.1014192.s001.tif]

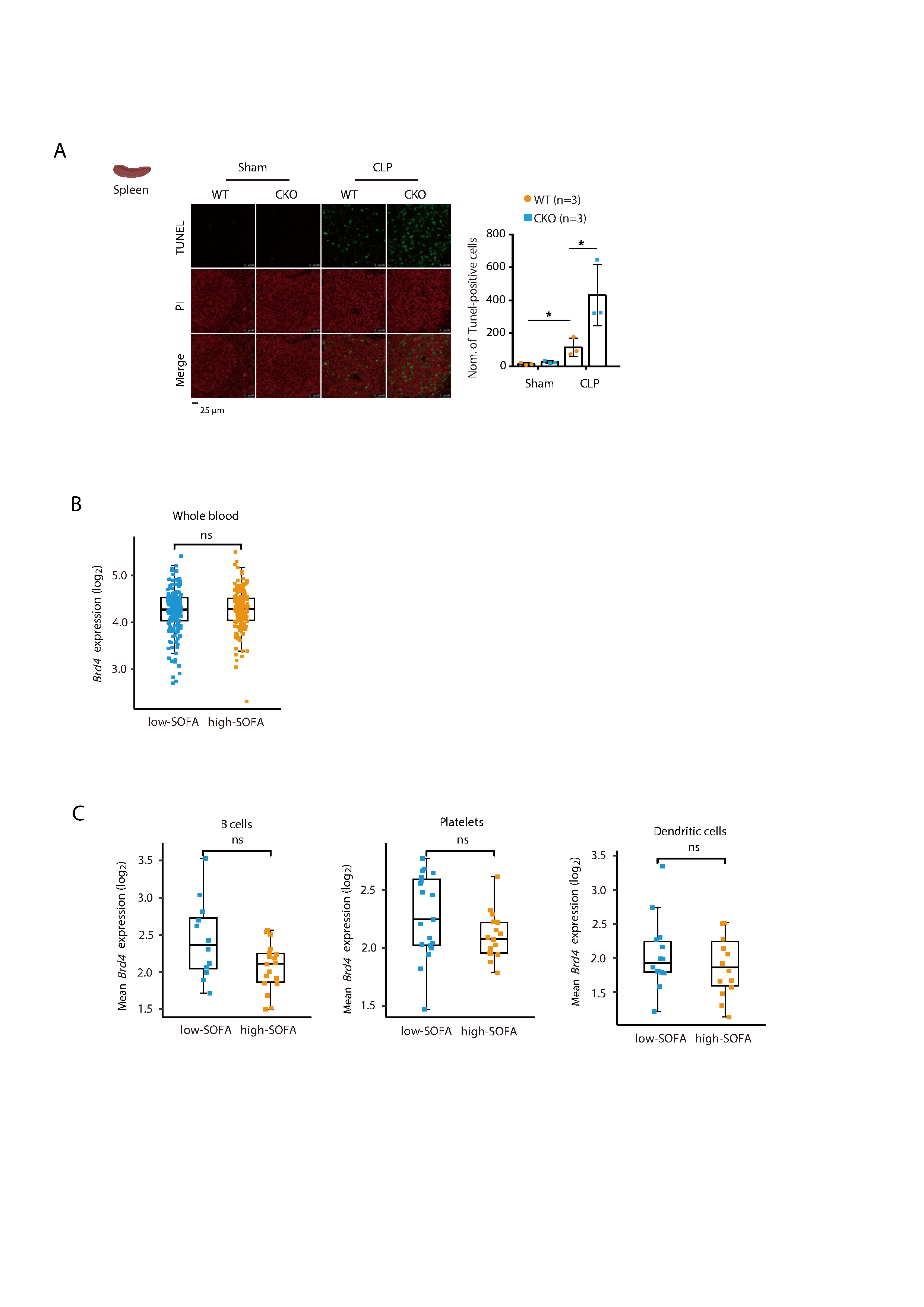

Supplement: S2 Fig — (A) Representative TUNEL-stained spleen tissue sections from WT and Brd4-CKO mice, 24 hours after sham or CLP surgery. (B) Comparison of BRD4 mRNA levels in whole blood between septic patients with low and high SOFA scores. Data source: GSE185263. (C) Comparison of BRD4 mRNA levels in B cells, platelets, and dendritic cells between low-SOFA and high-SOFA septic patients. Data source: SCP548. (TIF) [file ppat.1014192.s002.tif]

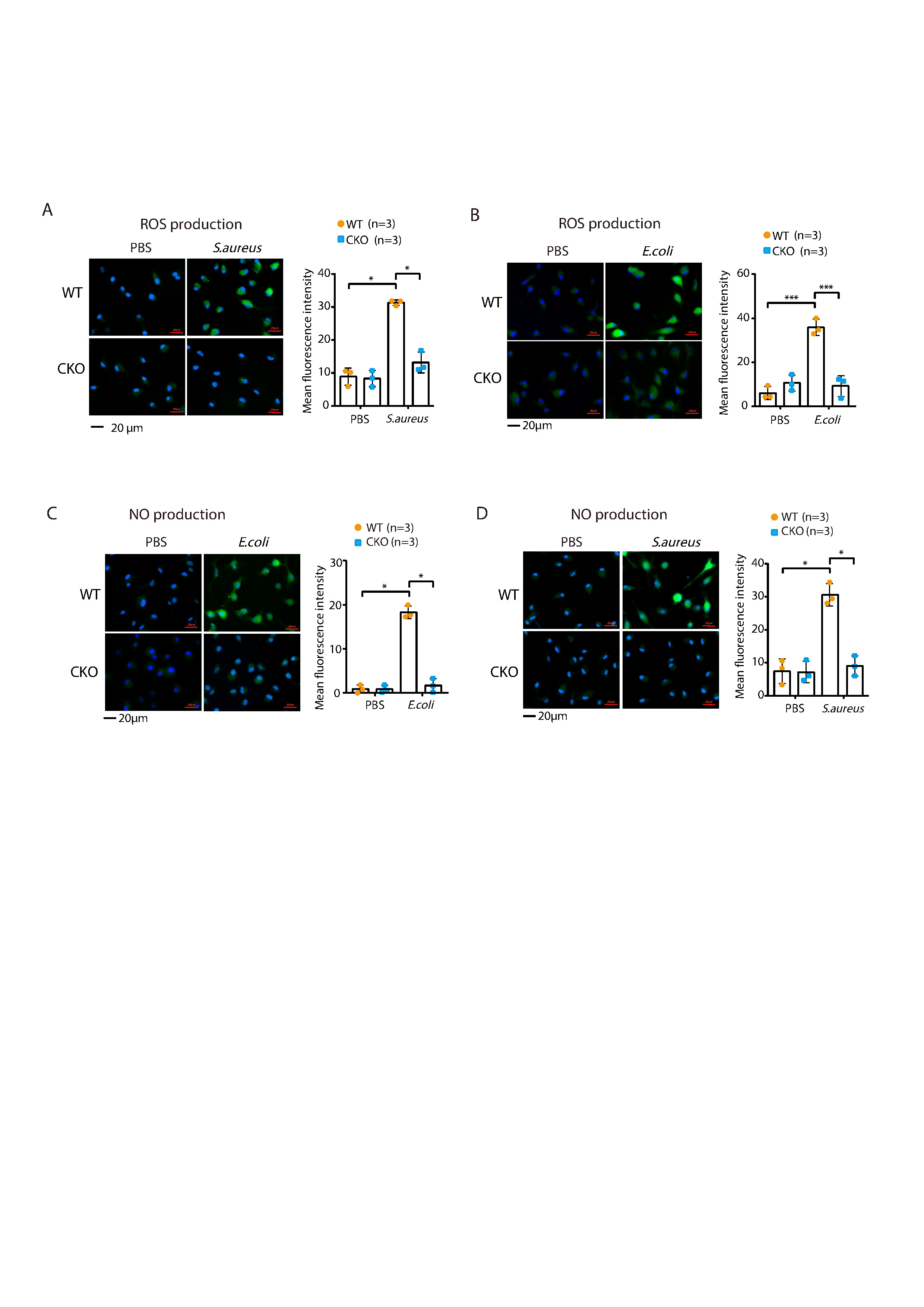

Supplement: S3 Fig — (A & B) WT and Brd4-deficient BMDMs were infected with E. coli (A) or S. aureus (B). Intracellular ROS levels were measured (n = 3). (C & D) WT and Brd4-deficient BMDMs were infected with E. coli (C) or S. aureus (D). Intracellular NO levels were measured (n = 3). (TIF) [file ppat.1014192.s003.tif]

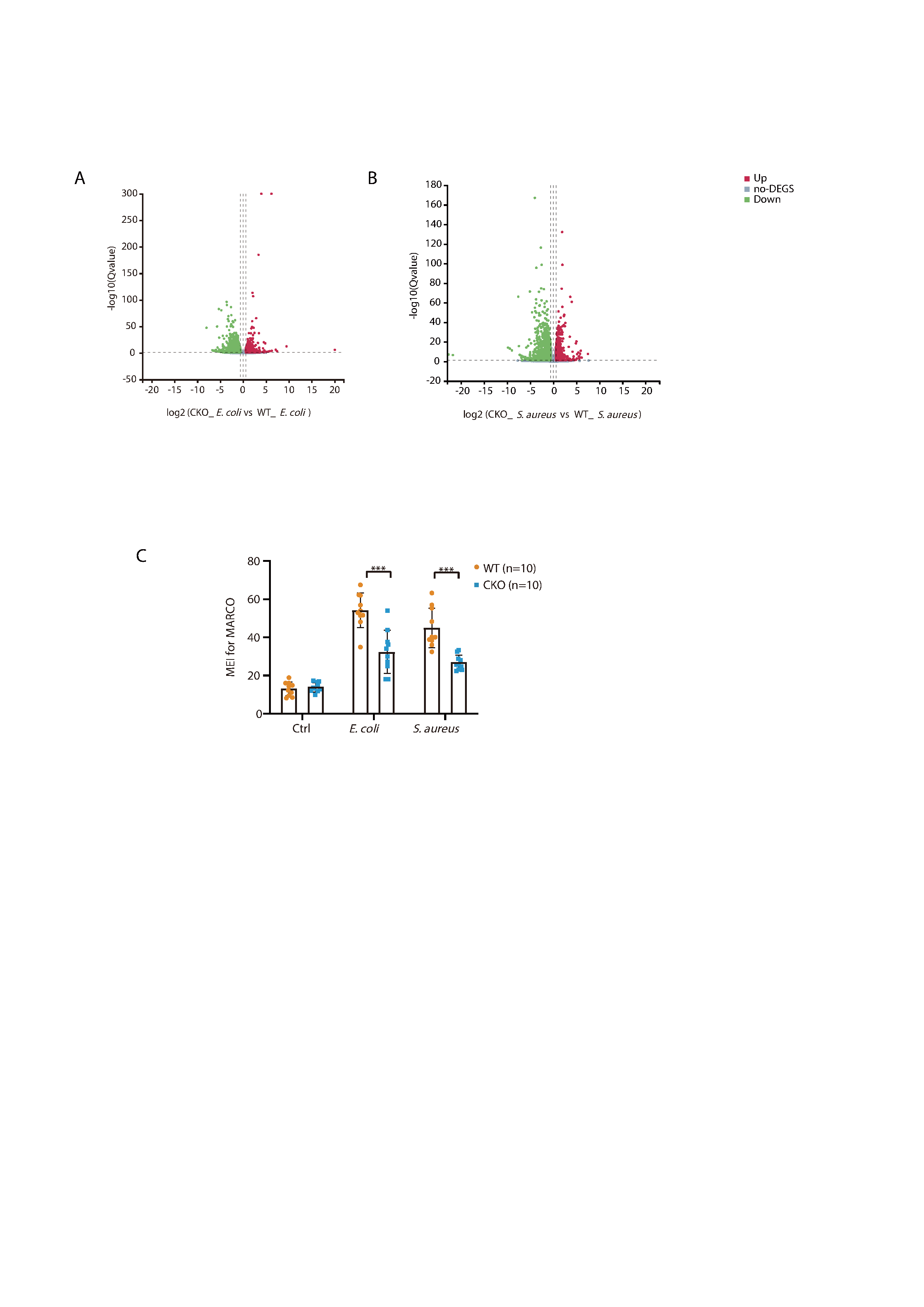

Supplement: S4 Fig — (A & B) Volcano plot of RNA-seq data comparing WT and Brd4-deficient BMDMs challenged with E. coli (A) or S. aureus (B). Upregulated genes in Brd4-deficient BMDMs relative to WT BMDMs are indicated by red dots, downregulated genes by green dots, and genes with no significant change by gray dots. (C) Quantification of MARCO protein levels in WT and Brd4-deficient BMDMs after infection with E. coli or S. aureus for 6 h, as assessed by immunofluorescence. (TIF) [file ppat.1014192.s004.tif]

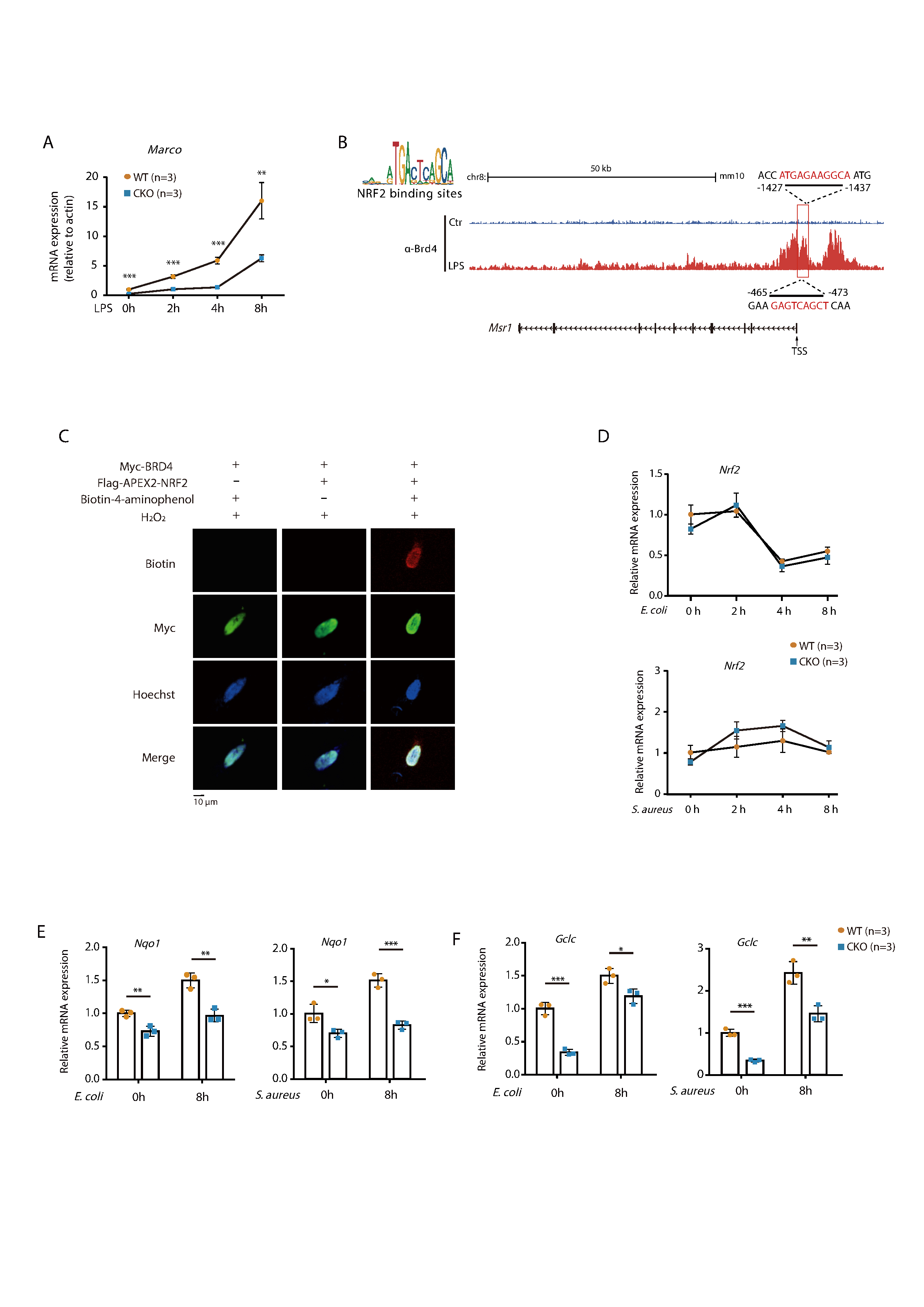

Supplement: S5 Fig — (A) WT and Brd4-deficient BMDMs were treated with or without LPS (10 ng/mL) for the indicated times. Marco mRNA levels were quantified by qRT-PCR. (B) Schematic of the potential binding sites of NRF2 on the murine Msr1 promoter region, identified by anti-BRD4 ChIP-seq. Data sources: GSE113226. (C) HEK293T cells were co- transfected with Myc-BRD4, with or without Flag-APEX2- NRF2, and treated with or without biotin-4-aminophenol (10 μM). After 30 min, H₂O₂ (1 mM) was added for stimulation, followed by immunofluorescence staining and confocal microscopy. (D-F) WT and Brd4-deficient BMDMs were treated with or without E. coli or S. aureus for the indicated times. Nrf2 (D), Nqo1 (E) and Gclc (F) mRNA levels were quantified by qRT-PCR. (TIF) [file ppat.1014192.s005.tif]

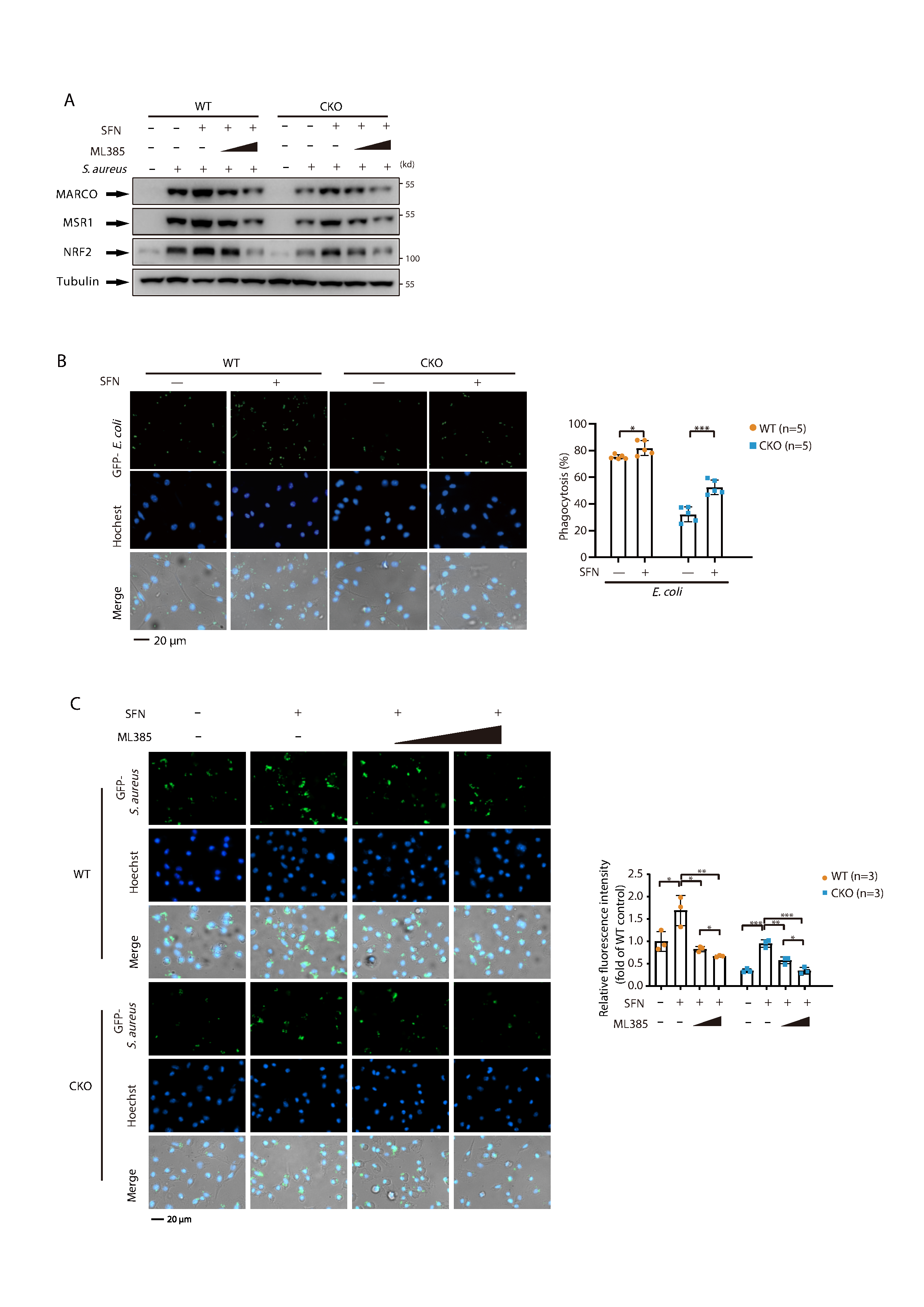

Supplement: S6 Fig — (A) WT and Brd4-deficient BMDMs pre-treated with or without the Sulforaphane (SFN, 10 μM) for 1 h, or with ML385 at two different concentrations: low (5 μM) and high (10 μM) for 12 h, prior to infection with S. aureus (MOI = 10) for 4 h. Protein levels of NRF2, MARCO, and MSR1 in cell lysates were assessed by immunoblotting. (B) WT and Brd4-deficient BMDMs were pre-treated with or without SFN for 1 h, followed by treatment with GFP-labeled E. coli for 1 h. Bacterial phagocytosis was quantified by fluorescence microscopy (n = 3). (C) WT and Brd4-deficient BMDMs pre-treated with or without the Sulforaphane (SFN, 10 μM) for 1 h or ML385 at two different concentrations: low (5um) and high (10um) for 12 h were infected with by S. aureus (MOI = 10) for 1 h. Bacterial phagocytosis was quantified by fluorescence microscopy (n = 3). (TIF) [file ppat.1014192.s006.tif]

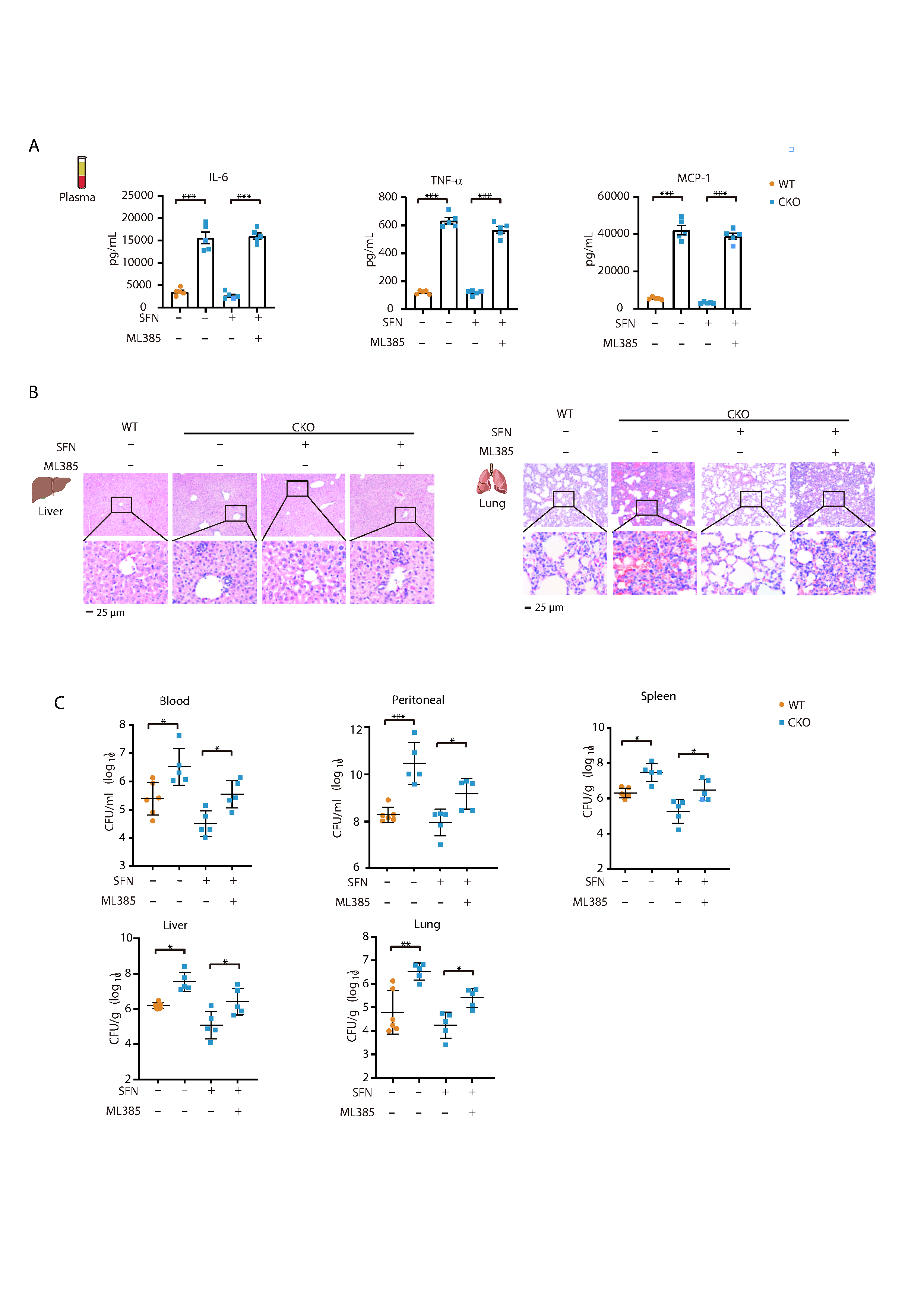

Supplement: S7 Fig — WT and Brd4-CKO mice were treated with SFN (0.4 mg/kg, i.v.) alone or in combination with ML385 (30 mg/kg, i.p.) and subjected to CLP. (A) Plasma levels of IL-6, TNF-α, and MCP-1 at 24 h post-CLP (n = 5 per group). (B) Representative H&E-stained sections of liver and lung from the indicated mice at 24 h post-CLP. (C) CFU in blood, peritoneal lavage fluid, lung, spleen, and liver at 24 h post-CLP (n = 5–6 per group). (TIF) [file ppat.1014192.s007.tif]

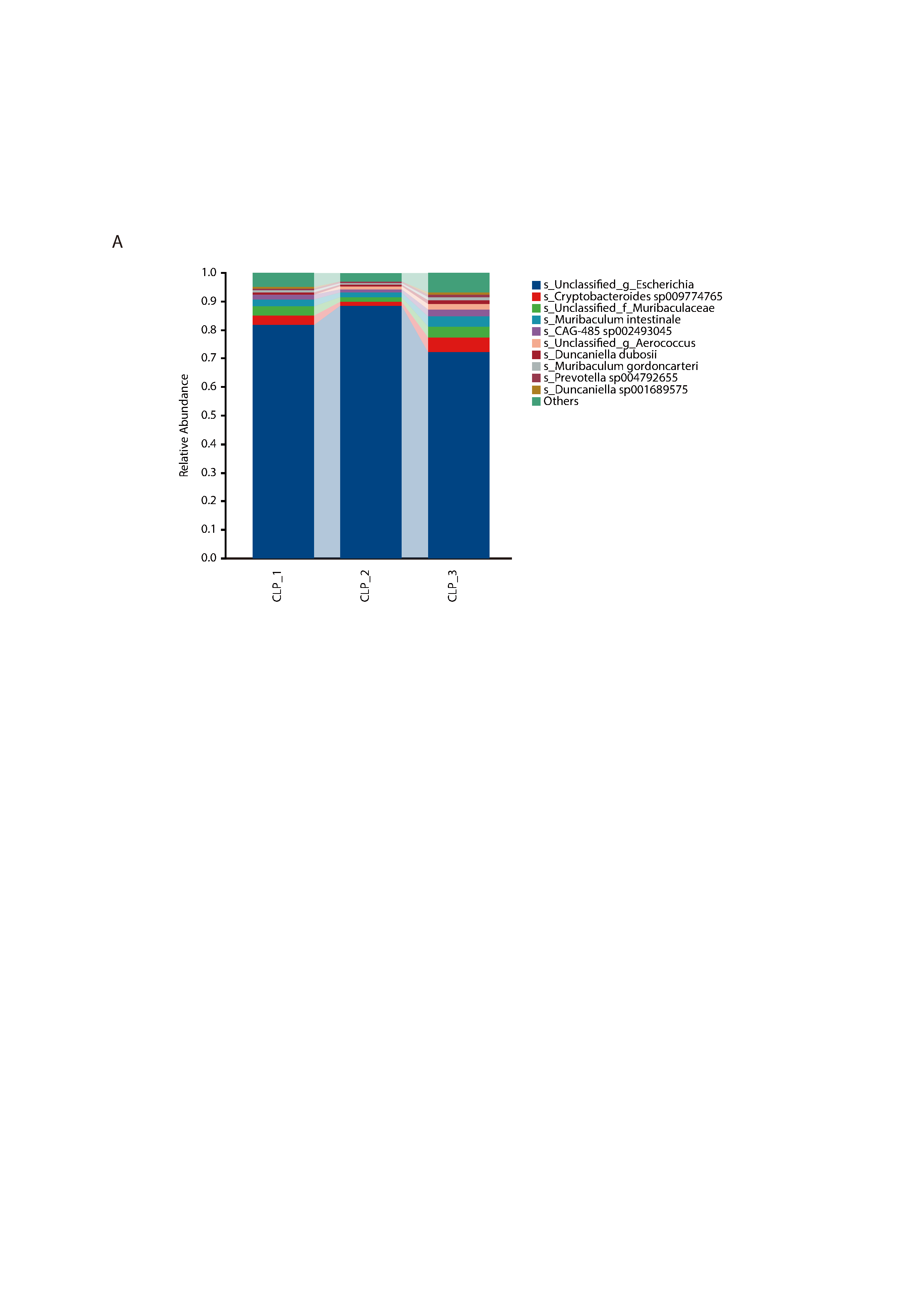

Supplement: S8 Fig — Taxonomic profiling of bacteria at the species level (n = 3). (TIF) [file ppat.1014192.s008.tif]

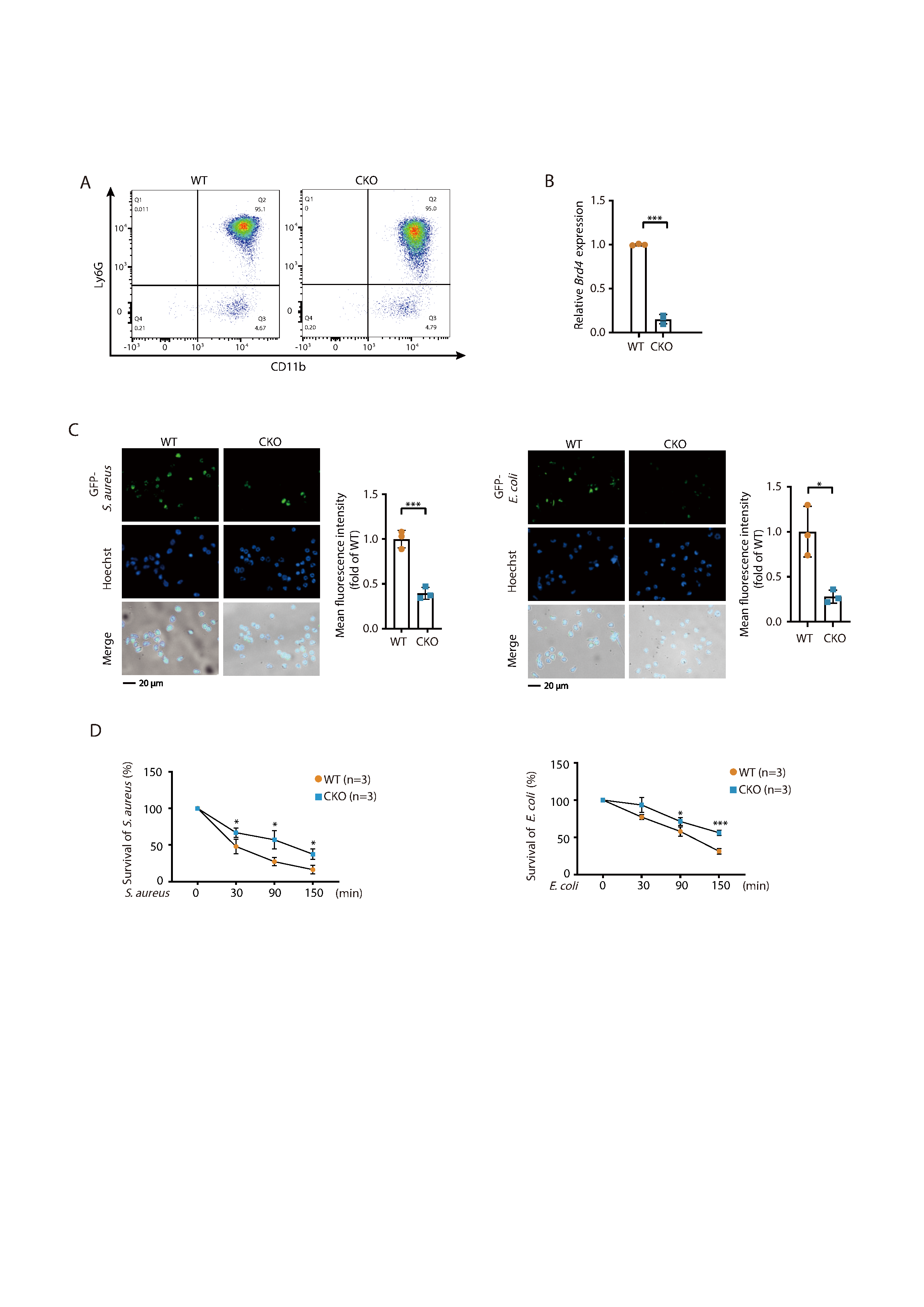

Supplement: S9 Fig — (A & B) Neutrophils isolated from the bone marrow of WT and Brd4-CKO mice were analyzed by flow cytometry for purity (A) and by RT-PCR for Brd4 mRNA levels (B). (C) WT and Brd4-deficient neutrophils were infected with GFP-labeled S. aureus (MOI = 10) or E. coli (MOI = 25) for 1 h. Phagocytosis was quantified by fluorescence microscopy (n = 3). (D) WT and Brd4-deficient neutrophils were infected with S. aureus (MOI = 10) or E. coli (MOI = 50), and bactericidal activity was assessed (n = 3). (TIF) [file ppat.1014192.s009.tif]

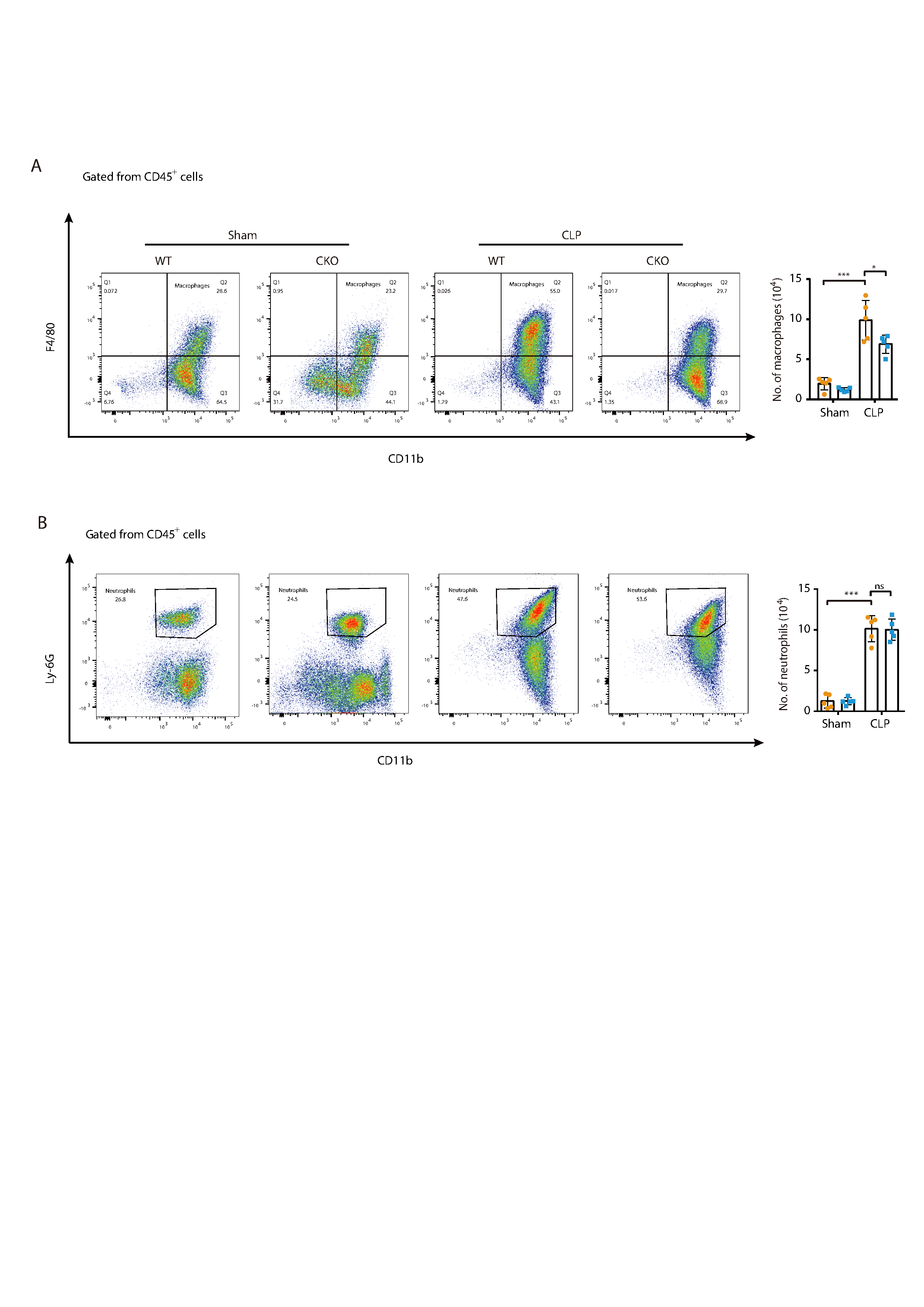

Supplement: S10 Fig — (A & B) Representative flow cytometry plots showing peritoneal macrophages (A) and neutrophils (B) from WT and Brd4-CKO mice, 24 hours after sham or CLP surgery (n = 5). (TIF) [file ppat.1014192.s010.tif]

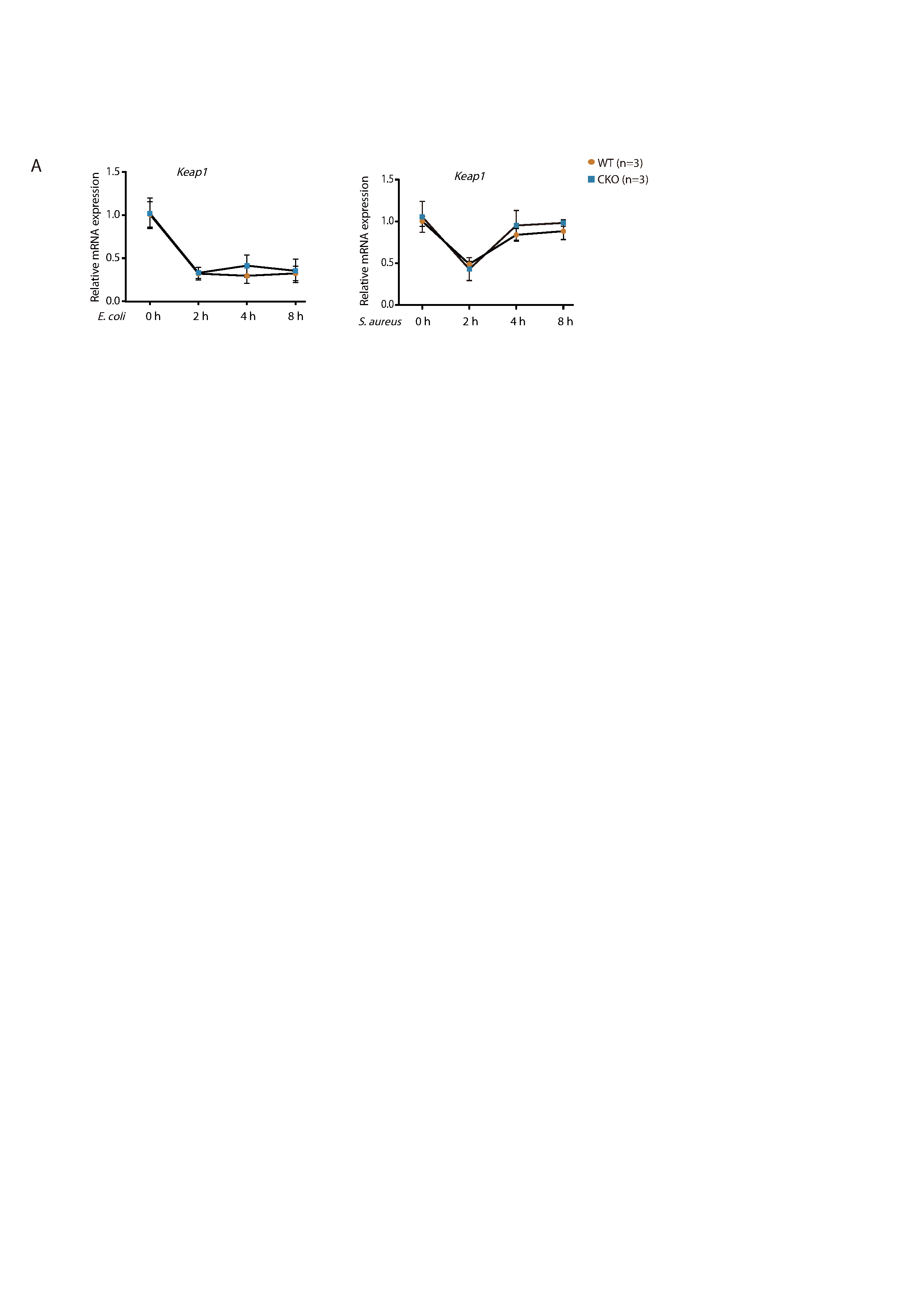

Supplement: S11 Fig — WT and Brd4-deficient BMDMs were infected with or without E. coli or S. aureus for the indicated times. Keap1 mRNA levels were quantified by qRT-PCR. (TIF) [file ppat.1014192.s011.tif]

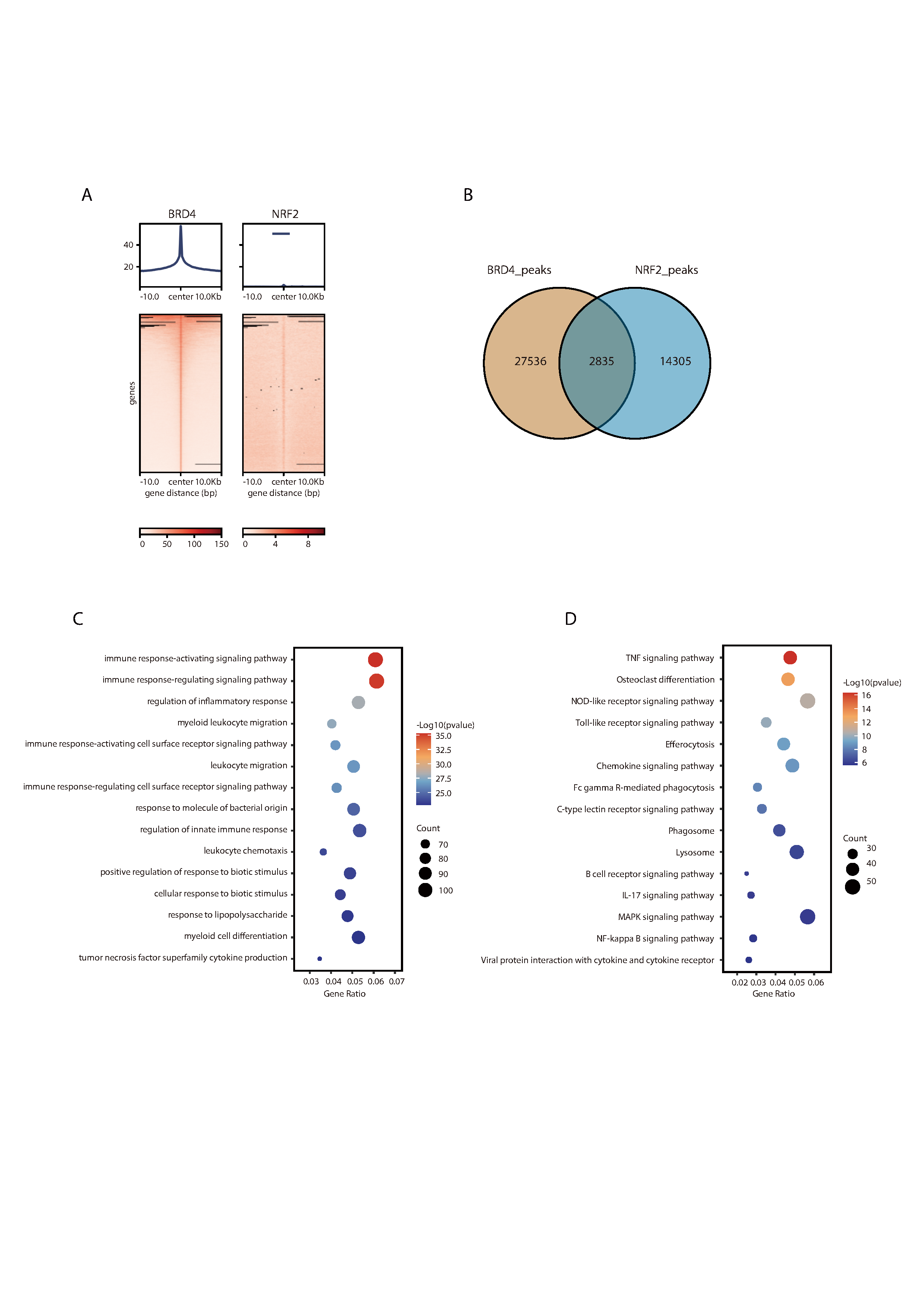

Supplement: S12 Fig — (A) Heatmaps depicting BRD4 and NRF2 binding signals in BMDMs based on published ChIP-seq datasets (BRD4: GSE113226; NRF2: DRA003771). Color scales indicate signal intensity. (B) Venn diagram showing genomic co-localization of BRD4 and NRF2. (C) Gene Ontology (GO) analysis of genes co-occupied by BRD4 and NRF2. (D) KEGG pathway analysis of genes co-occupied by BRD4 and NRF2. (TIF) [file ppat.1014192.s012.tif]

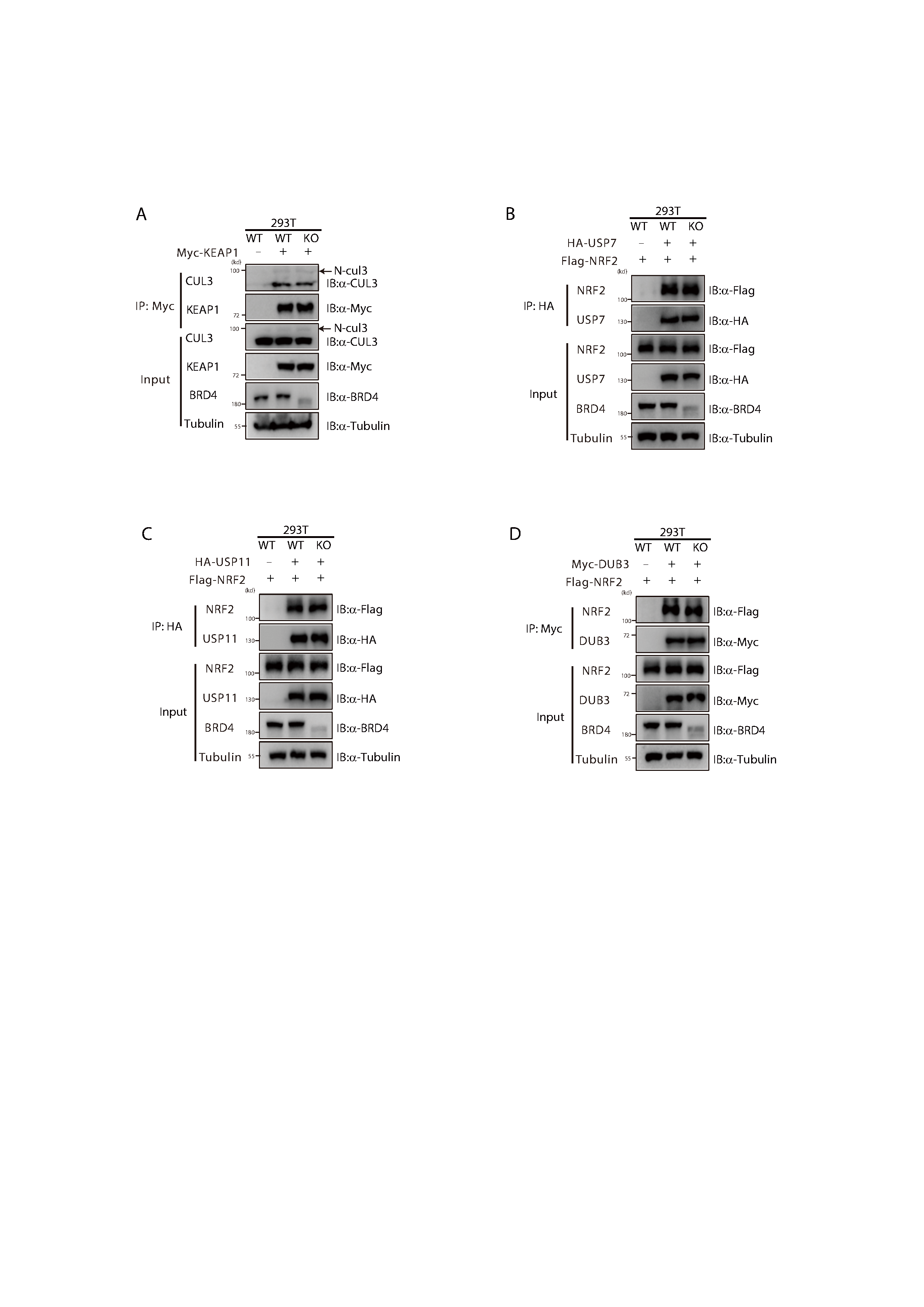

Supplement: S13 Fig — (A) WT and Brd4-knockout HEK293T cells expressing Myc-KEAP1 and immunoprecipitated with anti-Myc beads and analyzed by immunoblotting. (B & C) WT and Brd4-knockout HEK293T cells co-expressing HA-USP7 and Flag-NRF2 (B), HA-USP11 and Flag-NRF2 (C), were immunoprecipitated using anti-HA beads. The interaction was detected by immunoblotting. (D) WT and Brd4-knockout HEK293T cells co-transfected with Myc-DUB3 and Flag-NRF2 and cell lysates were immunoprecipitated with anti-Myc beads and analyzed by immunoblotting. (TIF) [file ppat.1014192.s013.tif]
