# Supplementary material for: BRD4 modulates antimicrobial defense via non-canonical NRF2 activation in macrophages to confer protection against sepsis
Source: PLoS Pathog. 2026 Apr 30;22(4):e1014192. doi: 10.1371/journal.ppat.1014192 (PMC13155688; doi:10.1371/journal.ppat.1014192)
Supplement: S1 Table — (DOCX) [file ppat.1014192.s014.docx]

| **S1 Table.** **Multivariable analysis of *BRD4* or MARCO expression in sepsis and healthy controls (adjusted for age and sex)** | | | |
| --- | --- | --- | --- |
| **CMAISE** |  |  |  |
| **Characteristic** | **OR** | **95% CI** | **p-value** |
| BRD4 | 0.28 | 0.18,0.41 | <0.001 |
| age | 1.04 | 1.02,1.05 | <0.001 |
| gender |  |  |  |
| F | - | - |  |
| M | 1.06 | 0.54,2.04 | 0.9 |
| Abbreviations: CI=Confidence Interval, OR=Odds Ratio | | | |
| **GSE95233** |  |  |  |
| **Characteristic** | **OR** | **95% CI** | **p-value** |
| BRD4 | 0.00 | 0.00, 0.00 | <0.001 |
| age | 1.03 | 0.97, 1.10 | 0.3 |
| gender |  |  |  |
| F | - | - |  |
| H | 32,615,313 | 0.00, | >0.9 |
| MARCO | 0.23 | 0.06,0.67 | 0.014 |
| age | 0.99 | 0.94,1.03 | 0.6 |
| gender |  |  |  |
| F | — | — |  |
| H | 15,258,406 | 0.00, | >0.9 |
| M | 0.21 | 0.04,0.85 | 0.035 |
| Abbreviations: CI=Confidence Interval, OR=Odds Ratio | | | |
