# Supplementary material for: BRD4 modulates antimicrobial defense via non-canonical NRF2 activation in macrophages to confer protection against sepsis
Source: PLoS Pathog. 2026 Apr 30;22(4):e1014192. doi: 10.1371/journal.ppat.1014192 (PMC13155688; doi:10.1371/journal.ppat.1014192)
Supplement: S2 Table — (DOCX) [file ppat.1014192.s015.docx]

| **S2 Table.** **Reagents and Resources** | | | |
| --- | --- | --- | --- |
| **Antibodies** | **Source** | **Identifier** | **Application** |
| BRD4 | Abmart | Antigen: hBRD4 (1063 ~ 1362 aa) | IP |
| BRD4 | Bethyl Lab | A301-985A100 | WB, ChIP, FACS |
| BRD3 | UpingBio | YP-Ab-16665 | WB |
| MARCO | Genetex | GTX39743 | FACS,IF,  WB |
| NRF2 | Proteintech | 16396-1-AP | WB, IF |
| KEAP1 | Proteintech | 10503-2-AP | WB |
| HA(3F10) | Roche | 11867423001 | WB |
| Lamin B1 | Abmart | P60054F | WB |
| Cullin 3 | Abcam | ab75851 | WB |
| c-Myc (9E10) | Santa Cruz | sc-40 | WB |
| Marco (for WB) | UpingBio | YP-Ab-05708 | WB |
| Monoclonal Anti-Flag (R) M2 | Sigma | F1804 | WB |
| Ubiquitin | Santa Cruz | sc-8017 | WB |
| β-Tubulin | Abbkine | A01030 | WB |
| Goat anti-rabbit IgG (H+L), (Alexa Fluor® 594) | Invitrogen | A-11012 | IF |
| FITC Goat Anti-Rat IgG(H+L) | Proteintech | SA00003-11 | IF |
| FITC anti-mouse CD45 30-F11 | BD Pharmingen | 553079 | FACS |
| PE anti-mouse CD45 | Biolegend | 103106 | FACS |
| Purified anti-mouse CD16/32 | Biolegend | 101302 | FACS |
| BV421 anti-mouse/human CD11b | Biolegend | 101236 | FACS |
| FITC anti-mouse/human CD11b | Biolegend | 101206 | FACS |
| APC anti-mouse F4/80 | Biolegend | 123116 | FACS |
| PerCP/Cyanine5.5 anti-mouse Ly-6G | Biolegend | 127615 | FACS |
| PerCP anti-human CD45 | Biolegend | 304026 | FACS |
| PE Donkey anti-rabbit IgG | Biolegend | 406421 | FACS |
| Donkey Anti-Rat IgG H&L (Alexa Fluor® 647) | Abcam | ab150155 | FACS |
| Fixable Viability Stain 780 | BD Pharmingen | 565388 | FACS |
| APC-R700 anti-mouse F4/80 | BD Pharmingen | 565787 | FACS |
| **Cell Lines and** [**Strains**](https://cn.bing.com/dict/search?q=Strains&FORM=BDVSP6&cc=cn) | |  |  |
| HEK293T | ATCC CRL-3216 | | |
| *Escherichia coli* | *ATCC 25922* | | |
| *Staphylococcus aureus* | NCTC 8325 | | |
| **Chemicals and Kits** | | |  |
| Quickchange site-directed mutagenesis kit | TOYOBO | KOD-401 | |
| BRD4 CRISPR/Cas9 KO | Santa Cruz | sc-400519-KO-2 | |
| Flag-NRF2 | Miaoling | P1629 | |
| Myc-KEAP1 | Miaoling | P18153 | |
| PCMV-USP7(human)-3xHA-Neo | Miaoling | P53761 | |
| pCMV-USP11(human)-2-3xHA-Neo | Miaoling | P41765 | |
| pEnCMV-USP17L2/DUB3(human)-3xMyc | Miaoling | P22730 | |
| Biotin-4-aminophenol | MCE | HY-141898 | |
| TUNEL Bright Green Apoptosis Detection Kit | Vazyme | A112 | |
| PrimeScript RT reagent Kit with gDNA Eraser | Takara | RR047A | |
| Trizol reagent | Invitrogen | 15596018 | |
| FS Universal SYBR Green Master | Vazyme | Q711-03 | |
| Reactive Oxygen Species Assay Kit | Beyotime | S0033S | |
| Hematoxylin and Eosin Staining Kit | Beyotime | C0105 | |
| Cycloheximide | MCE | HY-12320 | |
| MG132 | MCE | HY-13259 | |
| c-Myc Magnetic Beads | MCE | HY-K0206 | |
| Flag Magnetic Beads | MCE | HY-K0207 | |
| DAF-FM DA fluorophore | Beyotime | S0019S | |
| Hoechst 33342 | Beyotime | C1025 | |
| [Fluoromount-G Mounting Medium](https://www.southernbiotech.com/fluoromount-gr-0100-01) | Southern biotech | 0100-01 | |
| Sulforaphane | MCE | HY-13755 | |
| ML385 | MCE | HY-100523 | |
| Mouse Neutrophil Negative Selection Kit | MCE | HY-K0350 | |
| GST-tag protein purification kit | Beyotime | P2262 | |
| Murine M-CSF | Peprotech | 315-02 | |
| Phagocytosis Assay Kit (Green Zymosan) | Abcam | K397 | |
| Nuclear and Cytoplasmic Protein Extraction Kit | Beyotime | P0027 | |
| Mouse MCP-1 ELISA Kit | Biolegend | 432701 | |
| Mouse IL-6 ELISA Kit | Biolegend | 431301 | |
| Mouse TNF-α ELISA Kit | Biolegend | 430901 | |
| AST Assay Kit | Nanjing Jiancheng | C010-2-1 | |
| ALT Assay Kit | Nanjing Jiancheng | C009-2-1 | |
| CD11b Microbeads | Miltenyi biotec | 130-049-601 | |
| **Software and algorithms** |  |  |  |
| GraphPad Prism 9 | GraphPad Software, | https://www.graphpad.com | |
| FlowJo™ (version 10.8) | BD Biosciences | https://www.flowjo.com | |
| ImageJ | Image Software | https://imagej.net/ij | |
