# Supplementary material for: BRD4 modulates antimicrobial defense via non-canonical NRF2 activation in macrophages to confer protection against sepsis
Source: PLoS Pathog. 2026 Apr 30;22(4):e1014192. doi: 10.1371/journal.ppat.1014192 (PMC13155688; doi:10.1371/journal.ppat.1014192)
Supplement: S3 Table — (DOCX) [file ppat.1014192.s016.docx]

**S3 Table. Primers used for PCR**

| **qRT-PCR** | **Forward (5'-3')** | **Reverse (5'-3')** | |
| --- | --- | --- | --- |
| *Actin* | CGGTTCCGATGCCCTGAGGCTCTT | CGTCACACTTCATGATGGAATTGA | |
| *Brd4* | TGTTTAGAGTGCCTGGTGAA | GGTGGAGGATTGGTGCTG | |
| *Marco* | ATGGCACCAAGGGAGACAAAGG | GCCTGGTTTTCCAGCATCACCT | |
| *Msr1* | CGCACGTTCAATGACAGCATCC | GCAAACACAAGGAGGTAGAGAGC | |
| *Nqo1* | AGGATGGGAGGTACTCGAATC | AGGCGTCCTTCCTTATATGCTA | |
| *Gclc* | AGCTGCTCCCAACTGTGAG | TGAACGCCCACGAAAGCTAAA | |
| *Nrf2* | TCTTGGAGTAAGTCGAGAAGTGT | GTTGAAACTGAGCGAAAAAGGC | |
| *Keap1* | TGCCCCTGTGGTCAAAGTG | GGTTCGGTTACCGTCCTGC | |
| **ChIP** | | | |
| *Marco promoter* | TGTCTTCTGTGCTGCCCAAG | ACAGCCAGGAAACATTGTGC | |
| **gRNA** | | | |
| g*BRD4*-1 | ACTAGCATGTCTGCGGAGAG | | |
| g*BRD4*-2 | TGCCTGGACTATCATGATCT | | |
| g*BRD4*-3 | GGGAACAATAAAGAAGCGCT | | |
| **Deletion and mutation PCR primers** | | | |
|  | **Forward (5'-3')** | | **Reverse (5'-3')** |
| BRD4-ΔN | CCGCTCGAGGCTCCGAAGTCAAAAAAGAAGG | | GCTCTAGATCAGAAAAGATTTTCTTCAAATATTG |
| BRD4-ΔC | CGCGGATCCATGTCTGCGGAGAGCGGCCC | | CCGCTCGAGTTAAGCCATCTCTGTTTCGGAGTC |
| BRD4-ΔBD1 | GAGTGGTGCTCAAGACACTATTAATGGCAGAAGCTCTGGAAAAG | | TTCTGCCATTAATAGTGTCTTGAG |
| BRD4-ΔBD2 | CTCAAGGAGATGTTTGCCAAGGCCATGGCCCGCAAGCTCC | | GGCCATGGCCTTGGCAAAC |
| BRD4-ΔCTD | CCGCTCGAGATGTCTGCGGAGAGCGGCCC | | GCGGGGCCCTTAGGGTGAATGGTGGTGCTGGATGAC |
| BRD4-1-1279 aa | CCGCTCGAGATGTCTGCGGAGAGCGGCCC | | GCTCTAGATTATGCCTCCTCATGGGCCCGC |
| BRD4-1-1328 aa | CCGCTCGAGATGTCTGCGGAGAGCGGCCC | | GCTCTAGATTAGGCCAACTCCCTCTG |
| BRD4-1-1334 aa | CCGCTCGAGATGTCTGCGGAGAGCGGCCC | | GCTCTAGATTACTCCTGCTCCCGCTT |
| BRD4-3M | GAGTTGGCCATGATGATGGAGCAGGAGCGAAGACGCC | | CTCCTGCTCCATCATCATGGCCAACTCCCTCTGCTG |
| BRD4-4M | GAGCAGGAGATGATGATGATGGAAGCCATGGCAGCTAC | | CCATGGCTTCCATCATCATCATCTCCTGCTCCCGCTTCCG |
| NRF2-ΔN | CGGGGTACCATGGAATTCAATGACTCTGACTCT | | GCTCTAGACTAGTTTTTCTTTGTATCTGGCTTC |
| NRF2-ΔC | CGGGGTACCGCCACCATGATGGACTTGGAGTTGCC | | GCTCTAGATTACATTGTGCCTTCAGCGTGC |
| NRF2-ΔNeh2 | CGGGGTACCGCCACCCAGCCGGCCCAGCACATC | | GCTCTAGACTAGTTTTTCTTTGTATCTGGCTTC |
| NRF2-ΔDLG | CATCCTTTGGAGGCAAGACATAGTAAGTCGAGAAGTGTTTGAC | | CTCGACTTACTATGTCTTGCC |
| NRF2-ΔETGE | GCTCAGTTTCAACTGGATGAATTCCTCCCAATTCAGCCGGCC | | TTGGGAGGAATTCATCCAGTTG |
| PID | CCGGAATTCGCTGCGGTGGCTGCCGCCG | | CCGCTCGAGTTAGAAAAGATTTTCTTCAAATATTGACAATAGATCACTCTGG |
