# Supplementary material for: BRD4 modulates antimicrobial defense via non-canonical NRF2 activation in macrophages to confer protection against sepsis
Source: PLoS Pathog. 2026 Apr 30;22(4):e1014192. doi: 10.1371/journal.ppat.1014192 (PMC13155688; doi:10.1371/journal.ppat.1014192)
Supplement: S1 Text — (DOCX) [file ppat.1014192.s017.docx]

**S1 Text.**

**Detailed materials and methods**

***Patients and Sample Collection.*** Peripheral blood samples were collected from healthy controls and sepsis patients at The First Affiliated Hospital of Fujian Medical University (FJMU). Sepsis patients who met the clinical criteria for sepsis-3 (1) within 24 hours of admission to the Intensive Care Unit at The First Affiliated Hospital of FJMU were included in the study. This study was approved by the Ethics Committee of FJMU (ethical approval number: 2023-167). Informed consent was obtained from all participants in written form.

***Mice.*** Myeloid-cell-specific *Brd4* knockout mice were generated by crossing *Brd4* floxed mice with LysM-Cre mice, as previously described (2). *Brd4* floxed mice littermates were used as WT controls. Male mice, aged 8–10 weeks, were used for experiments. Mice were housed under specific pathogen-free conditions with a constant temperature of 23°C, humidity of 65%, and a 12-hour light/dark cycle. They had *ad libitum* access to food and water. All experimental procedures were reviewed and approved by the Institutional Animal Care and Use Committee of FJMU (ethical approval number: 2023-Y-1132).

***Cell lines, antibodies, and plasmids construction*.** HEK293T cells were obtained from ATCC. The HEK293T *BRD4*-KO cell line was generated using CRISPR/Cas9-mediated gene editing with a targeting plasmid (sc-400519-KO-2, Santa Cruz Biotechnology) transfected into HEK293T cells. Following transfection, cells were sorted by flow cytometry (FACSAria III, BD). Single-cell clones were isolated, expanded, and screened for the complete loss of BRD4 protein expression via immunoblotting and DNA sequencing.

The Myc-BRD4 and HA-Ubiquitin expression constructs were described previously (3). Plasmids encoding Flag-NRF2 and Myc-KEAP1 were obtained from Miaoling Biotechnology, with catalog numbers P1629 and P18153, respectively. Mutants of BRD4 and NRF2 were generated using the QuickChange site-directed mutagenesis kit (KOD-401, TOYOBO). All plasmid sequences were confirmed by DNA sequencing.

Detailed information regarding antibodies and other reagents is provided in Supplementary Table 2.

***Protein Expression and Purification.*** The GST-PID (1302-1362aa) fusion protein was expressed in *E. coli* BL21. Bacterial culture was induced with 1 mM isopropyl β-D-1-thiogalactopyranoside (IPTG) at 25°C overnight. The recombinant protein was purified using GST-tag protein purification kit (Beyotime, P2262),

***Cecal ligation and puncture.*** Mice were anesthetized with isoflurane in oxygen and positioned in a dorsal recumbent posture. After shaving and aseptic preparation of the ventral surface using 70% ethanol, a ∼1 cm midline incision was made to exteriorize the cecum. Once identified, the cecum was ligated with 3-0 silk thread approximately 1 cm distal to the tip, then perforated twice with a 22G needle for moderate sepsis or a 19G needle for severe sepsis on the antimesenteric surface. A small amount of fecal material was subsequently extruded. Sham-operated mice underwent the same surgical procedure, including laparotomy and cecal exteriorization, but without ligation or perforation. Mice were euthanized 24 hours post-surgery for tissue collection. Survival was monitored every 6 hours for the first 48 hours, and then every 12 hours for up to 8 days. SFN or ML385-treated mice received an intravenous injection of sulforaphane (SFN) (0.4 mg/kg) and intraperitoneal administration of ML385 (30 mg/kg) for 24 hours prior to the surgical procedure, followed by daily intravenous doses for three consecutive postoperative days.

***In Vivo AAV Delivery****.* AAV9-Lyz2-Vector or AAV9-Lyz2-*Nrf2* (WZ Biosciences, Shandong, China) was administered to WT and *Brd4* knockout mice at 5 × 10¹¹ vg in 100 μL per mouse via intravenous injection. CLP was performed three weeks after viral delivery.

**Quantification of inflammatory cytokines*.*** Inflammatory cytokines were quantified in plasma and organ supernatants (lung and spleen). Lung and spleen homogenates were prepared using a Precellys 24 homogenizer in lysis buffer containing 1 mM EDTA, 1 mM PMSF, and 1 mM DTT (25 mL PBS per 40 mg tissue). The supernatants were collected following triple centrifugation (6,600 rpm for 20 seconds at 4°C). Cytokine levels were quantified using the BD CBA Mouse ELISA Kit for a multiplex analysis of IL-6, TNF-α, and MCP-1.

**Quantification of ALT and AST*.*** Heparinized blood obtained via cardiac puncture was centrifuged, and the levels of alanine transaminase (ALT) and aspartate transaminase (AST) were quantified using commercial kits (Nanjing Jiancheng) following the manufacturer's protocols. Measurements were performed using a microplate reader (Thermo Fisher Scientific).

***Histology and tissue staining.*** At designated postoperative time points, mice were euthanized, and tissues were harvested for histological analysis. The liver, lung, and spleen were fixed, sectioned at a thickness of 5 μm, and stained with hematoxylin and eosin (H&E). Lung injury was assessed by a blinded observer using a scoring system ranging from 0 to 3 (0 = no injury, 1 = mild, 2 = moderate, 3 = severe injury), based on the following criteria: exudate formation, hyperemia/congestion, neutrophil infiltration, hemorrhage, debris, and hyperplasia. The individual scores for each group were summed, and group mean scores were plotted as bar graphs.

***Myeloperoxidase (MPO) assays.*** Lung tissues (20–30 mg) were snap-frozen in liquid nitrogen and powdered. The tissue powder was then homogenized in 200 μL of 50 mM potassium phosphate buffer containing 0.5% hexadecyltrimethylammonium bromide (A600108, Sangon Biotech) using a homogenizer while maintaining the samples on ice. After centrifugation, the supernatant was diluted in a reaction solution containing 0.167 mg/mL O-Dianisidine dihydrochloride (D1657, Tokyo Chemical Industry) and H₂O₂ (A001896, Sangon Biotech) as substrates. Absorbance was measured at 450 nm using a spectrophotometer, and readings were taken at 30-second intervals. MPO activity was calculated using the following formula: [ΔA(t2-t1)]/Δmin x (1.13 x 10-2)^2^.

***Determination of bacterial colony-forming units (CFUs).*** Peripheral blood and peritoneal lavage fluid from mice were collected and subjected to serial dilution before plating on blood agar plates. Concurrently, liver, lung, and spleen tissues were excised, and homogenates were prepared by processing 50 mg of tissue in 500 μL of phosphate-buffered saline. The homogenates were then serially diluted. Aliquots (100 μL) of each dilution were plated onto blood agar plates and incubated under aerobic conditions overnight at 37°C (4). After 24 hours of incubation, bacterial colonies were counted to determine the CFUs.

***Flow cytometric analysis.*** Flow cytometry was performed using an LSRFortessa X-20 (BD Biosciences). Cells (1 × 10^6^ per sample) were pre-incubated with 0.4 μg of anti-mouse CD16/32 antibody to block Fc receptors, followed by a 30-minute incubation with fluorochrome-conjugated antibodies at 4°C in the dark. Antibody details are provided in Supplementary Table 2. Gating strategies were as follows: CD45^+^ CD11b^+^ F4/80^+^ cells as mouse peritoneal macrophages, CD45^+^ CD11b^+^ Ly6G^+^ cells were identified as mouse peritoneal neutrophils, and CD45^+^ CD11b^+^ cells as human peripheral blood monocytes. Data analysis was performed using FlowJo software.

***Preparation of BMDMs and Infections.*** BMDMs were generated according to established protocols (5). Bone marrow cells were aseptically isolated from the tibiae and femora of male C57BL/6 mice (8–10 weeks old). The isolated cells were cultured in complete DMEM/F12 medium, supplemented with 10% heat-inactivated fetal bovine serum (Gibco, 10270106), 1% penicillin-streptomycin (Hyclone, SV30010), 2 mM L-glutamine (Gibco, 25030081), 10 mM HEPES buffer (Gibco, 15630080), and 20 ng/mL recombinant murine macrophage colony-stimulating factor (PeproTech, 315-02). The culture medium was replaced on day 4. Differentiated BMDMs were harvested on day 7 for subsequent experimental procedures. For infection experiments, BMDMs were exposed to either *E. coli* or *S. aureus* at a multiplicity of infection (MOI) of 10, unless otherwise specified.

***Preparation of neutrophils and Infections.*** Bone marrow cells were aseptically isolated from the tibiae and femora of male C57BL/6 mice (8–10 weeks old). Subsequently, neutrophils were isolated using a commercial mouse neutrophil negative selection kit (MCE, HY-K0350), and their purity was assessed by flow cytometry. For infection, the isolated neutrophils were allowed to adhere to fibronectin-coated plates prior to exposure to either *E. coli* or *S. aureus.*

***Phagocytosis assays.*** For in vivo experiments, WT and *Brd4*-CKO mice were administered intraperitoneal injections of 1 × 10⁷ GFP-labeled *E. coli* or *S. aureus*. Animals were euthanized 15 minutes post-injection. The efficiency of bacterial phagocytosis by peritoneal macrophages was analyzed by flow cytometry.

For in vitro studies, WT and *Brd4*-deficient BMDMs and neutrophils were infected with GFP-labeled *E. coli* (MOI 25) or *S. aureus* (MOI 10) at 37°C for 1 hours. After PBS washes to remove unbound bacteria, extracellular bacteria were eliminated using gentamicin treatment (300 μg/mL, 1 hour at 37°C). The cells were then fixed in 4% formaldehyde and stained with Hoechst 33342 (Beyotime) for nuclear counterstaining. The efficiency of bacterial phagocytosis by BMDMs was analyzed by fluorescence microscopy.

The phagocytosis of zymosan particles by BMDMs was assessed using the EZCell™ system (BioVision, K397) following the manufacturer's instructions.

***Electron microscopy.*** BMDMs were pulsed with zymosan (20 particles per cell) at 37°C for 30 minutes. For transmission electron microscopy (TEM), cells were plated on bacteriological plastic and subjected to primary fixation with 3% glutaraldehyde, 1.5% paraformaldehyde and 0.1% picric acid in 100 mM cacodylate buffer (pH 7.0). Post-fixation was performed with 1% osmium tetroxide and 1.5% potassium ferrocyanide in cacodylate buffer at 4°C for 1.5 hours, followed by en bloc staining with 2% aqueous uranyl acetate at 4°C for 2 hours in the dark. After fixation, cells were dehydrated through a series of ethanol solutions, treated with propylene oxide for detachment, and embedded in epoxy resin. Ultrathin sections (90 nm) were stained with uranyl acetate and lead citrate and examined using a FEI Tecnai G2 transmission electron microscope.

For scanning electron microscopy (SEM), zymosan-treated BMDMs on coverslips underwent the same fixation procedure. Following fixation, the samples were subjected to critical point drying and coated with 10 nm of gold before imaging on a FEI QUANTA 450 scanning electron microscope.

***Bacterial Killing assays.*** BMDMs and neutrophils were infected with *E. coli* (MOI = 50) or *S. aureus* (MOI = 10) in serum-free DMEM at 37°C for 30 minutes. Extracellular bacteria were removed by three PBS washes, followed by elimination of residual bacteria using 300 μg/mL gentamicin (37°C, 60 minutes). At specified post-infection time points (0, 30, 90, or 150 minutes), cell lysis was performed using 0.2% (v/v) Triton X-100. Serial dilutions of the cell lysates were plated on blood agar, and CFUs were enumerated after overnight incubation. CFU counts at 0 minutes (t = 0) served as the 100% reference.

***qRT-PCR.*** Total RNA from cells or tissues was isolated using the Trizol reagent (15596018, Invitrogen) and was reverse-transcribed using the PrimeScript RT reagent Kit with gDNA Eraser (RR047A, Takara Biomedical Technology). Gene expression was analyzed with FS Universal SYBR Green Master (Q711-03, Vazyme Biotech, Nanjing). Primer sequences are provided in Supplementary Table 3.

***RNA-seq.*** Following 4-hour stimulation with or without *E. coli* or *S. aureus*, total RNA was extracted from WT and *Brd4*-deficient BMDMs. RNA quality was performed using an Agilent 2100 Bioanalyzer. Libraries were sequenced on the DNBSEQ platform (Beijing Genomics Institute). Clean reads were aligned to the GRCm38.p6 mouse reference genome (Assembly GCF_000001635.26) using HISAT2. Gene expression was quantified as FPKM values. Differentially expressed genes (DEGs) were defined by |log₂ (fold change) | ≥ 1 with false discovery rate (FDR)-adjusted Q-value ≤ 0.05. Functional annotation of DEGs was conducted through Gene Ontology (GO) and Kyoto Encyclopedia of Genes and Genomes (KEGG) pathway analyses using the DAVID database.

***Chromatin immunoprecipitation assays (ChIP).*** Chromatin immunoprecipitation was performed according to established methods (5). BMDMs (1 × 10⁷ cells) were fixed with 1% formaldehyde and quenched with 125 mM glycine. Chromatin was fragmented to approximately 500 bp by sonication. Antibody-specific immunoprecipitation proceeded overnight at 4°C. After reversing crosslinks with proteinase K (0.2 mg/mL, 65°C, overnight), DNA was purified using Qiagen spin columns and analyzed by quantitative PCR. Primer sequences are provided in Supplementary Table 2.

***Immunoprecipitation.*** HEK293T cells were lysed in lysis buffer (30 mM HEPES pH 7.9, 0.3 M NaCl, 1.5 mM MgCl₂, 0.4 mM EDTA, 20% glycerol, 0.5% NP-40) supplemented with freshly added 1 mM DTT and 1 mM PMSF. Cells were disrupted by 30-min vortex-lysis at 4°C followed by clarification (13,000 g, 10 min, 4°C). The supernatant was adjusted to a final NaCl concentration of 0.15 M and glycerol concentration of 10%, then pre-cleared with protein A/G agarose beads. Protein complexes were enriched by Myc magnetic beads or anti-Flag beads for 2 hours at 4°C with constant agitation. Subsequently, the protein-bound beads were pelleted by centrifugation, washed extensively, and analyzed by western blotting.

***Ubiquitination analysis.*** To assess the ubiquitination of exogenous NRF2, HEK293T cells were transfected with the indicated plasmids and treated with MG132 (10 μM) for 4 hours prior to lysis. At 48 hours post-transfection, cells were washed with PBS and lysed in cell lysis buffer (10 mM Tris-HCl, pH 8.0, 1% SDS, 150 mM NaCl). Lysates were heated at 95°C and then diluted with nine volumes of dilution buffer (10 mM Tris-HCl, pH 8.0, 150 mM NaCl, 2 mM EDTA, 1% Triton X-100, 1× protease inhibitor). After a 30-minute incubation at 4°C, the samples were centrifuged at 18,000 × g for 30 minutes at 4°C. The resulting supernatant was subjected to immunoprecipitation using an anti-Flag antibody, followed by western blot analysis.

To assess the ubiquitination of endogenous NRF2, WT and *Brd4*-deficient BMDMs were lysed following a 6-hour infection with *S. aureus* (MOI = 10). BMDMs were similarly treated with MG132 for 4 hours prior to cell lysis. The lysates were denatured as described above, immunoprecipitated with an anti-NRF2 antibody, and analyzed by western blotting.

***Protein half-life analysis.*** WT and *Brd4*-deficient BMDMs were infected with Escherichia coli (MOI = 25) for 2 hours or Staphylococcus aureus (MOI = 10) for 6 hours, respectively. Following infection, cells were treated with cycloheximide (CHX) at a final concentration of 5 μg/mL at the indicated time points. Protein stability was assessed by western blot analysis.

***Immunofluorescence.*** BMDMs (7 × 10^4^ cells/well) were cultured on sterile 14-mm glass coverslips and infected *E. coli* (MOI = 25) or *S. aureus* (MOI = 10) for 6 hours. Following infection, cells were fixed with 4% paraformaldehyde in PBS for 20 min, washed three times with PBS, and permeabilized with 0.5% (v/v) Triton X-100 in PBS (pH 7.4) for 1 h at room temperature. Samples were blocked with goat serum for 1 hour at 37 ℃. After blocking, cells were incubated overnight at 4°C with gentle agitation using the following primary antibodies: Rat anti-MARCO (1:100, Genetex) and Rabbit anti-NRF2 (1:100, Proteintech). The next day, after three PBS washes, the samples were incubated with the corresponding secondary antibodies: anti-rabbit IgG Alexa Fluor 594 (1:1000, Invitrogen), anti-rat IgG Alexa Fluor 488 (1:1000, Proteintech), and Hoechst 33342 (Beyotime) for 1 hour at room temperature. Samples were mounted with an anti-fade mounting medium (Southern Biotech) and imaged using a confocal microscope.

***Nuclear-Cytoplasmic Fractionation.*** BMDMs were seeded into 6-well tissue culture plates at 1.2 × 10⁶ cells/well in DMEM/F12 complete medium supplemented with 10% FBS. Cells were challenged with *S. aureus* (MOI =10) for 6 hours. Following infection, cells were collected in PBS, centrifuged to collect the cell pellet, and then nuclear/cytoplasmic fractions were isolated using the nuclear and cytoplasmic protein extraction kit (P0027, Beyotime) following manufacturer's instructions. Finally, samples detected by western blotting.

***Collection of bulk RNA and single-cell RNA-seq data.*** One sepsis-related microarray datasets, GSE95233, was retrieved from the Gene Expression Omnibus (GEO) database (https://www.ncbi.nlm.nih.gov/geo/). Additionally, the bulk RNA-seq and single-cell RNA-seq (scRNA-seq) datasets from the Chinese Medical Alliance for Sepsis (CMAISE, Project ID: PRJCA006118; https://ngdc.cncb.ac.cn/) were also included. The raw microarray data were preprocessed by background correction and log2 transformation, followed by quantile normalization using the Robust Multi-array Average (RMA) algorithm. For the CMAISE RNA-seq data, gene expression values were obtained as normalized counts and log2-transformed prior to downstream analysis. For each sample, if multiple probes corresponded to the same Gene ID, their expression values were averaged to generate a single representative value per gene.

Single-cell RNA sequencing (scRNA-seq) datasets from septic patients were obtained from the GEO database (GSE167363), the Single Cell Portal (SCP548), and CMAISE. The scRNA-seq data were processed and analyzed using R software and the ‘Seurat’ package (6). To minimize disease-stage heterogeneity and ensure a standardized baseline for clinical comparison, all patients level analyses were strictly performed using samples collected on the first day of sepsis diagnosis. Data from Days 1, 3, and 5 were utilized exclusively for the longitudinal analysis to characterize the temporal dynamics of gene expression.

***Dimensionality reduction, clustering, and cell-type annotation.*** Highly variable genes were identified using the "FindVariableFeatures" function, followed by principal component analysis (PCA) on these genes. Batch effects across samples were corrected using the "RunHarmony" function from the Harmony package (7). Cell clustering was performed using the "FindClusters" function, utilizing the same principal components (PCs) as those used in the "RunUMAP" dimensionality reduction. Cell types were assigned to clusters based on their canonical marker expression: B cells (MS4A1, CD79A), monocytes (CD14, VCAN), T cells (CD3D), dendritic cells (DCs) (FCER1A, CST3, GZMB), neutrophils (ITGAM, FCGR3B, SELL), natural killer (NK) cells (GNLY, NKG7), plasma cells (SDC1, CD79A, JCHAIN, GHA1), erythroid cells (ALAS2), and platelets (PPBP, GP9) (8).

***Calculation of target gene expression in cell subpopulations.*** Using monocytes as an example, monocytes were first extracted from the single-cell expression matrix. Target genes were filtered based on an expression threshold (normalized counts > 0), excluding cells with undetected expression (technical zeros) while retaining those with detectable gene expression. For each gene, the mean normalized expression value across the monocytes was calculated to represent its expression level in the monocyte subpopulation.

***Statistics.*** Omics data obtained from public databases are presented as medians ± interquartile range and were analyzed using the Wilcoxon rank-sum test (two-tailed). Boxplots display the 25% (lower hinge), 50% (median), and 75% quantiles (upper hinge) percentiles. Other experimental data were expressed as mean ± standard deviation (SD). Comparisons between two groups were conducted using an unpaired two-tailed Student’s t-test, while comparisons involving more than two groups utilized one-way or two-way analysis of variance (ANOVA). A p-value ≤ 0.05 was considered statistically significant. Statistical significance is indicated as follows: *p < 0.05, **p < 0.01, ***p < 0.001. ns, not significant.

**References**

1. Singer M, Deutschman CS, Seymour CW, Shankar-Hari M, Annane D, Bauer M, et al. The Third International Consensus Definitions for Sepsis and Septic Shock (Sepsis-3). JAMA. 2016;315(8):801-10.

2. Bao Y, Wu X, Chen J, Hu X, Zeng F, Cheng J, et al. Brd4 modulates the innate immune response through Mnk2-eIF4E pathway-dependent translational control of IkappaBalpha. Proc Natl Acad Sci U S A. 2017;114(20):E3993-E4001.

3. Hu X, Dong SH, Chen J, Zhou XZ, Chen R, Nair S, et al. Prolyl isomerase PIN1 regulates the stability, transcriptional activity and oncogenic potential of BRD4. Oncogene. 2017;36(36):5177-88.

4. de Tymowski C, Heming N, Correia MDT, Abbad L, Chavarot N, Le Stang MB, et al. CD89 Is a Potent Innate Receptor for Bacteria and Mediates Host Protection from Sepsis. Cell Rep. 2019;27(3):762-75 e5.

5. Hu X, Dong X, Li G, Chen Y, Chen J, He X, et al. Brd4 modulates diet-induced obesity via PPARgamma-dependent Gdf3 expression in adipose tissue macrophages. JCI Insight. 2021;6(7).

6. Stuart T, Butler A, Hoffman P, Hafemeister C, Papalexi E, Mauck WM, 3rd, et al. Comprehensive Integration of Single-Cell Data. Cell. 2019;177(7):1888-902 e21.

7. Korsunsky I, Millard N, Fan J, Slowikowski K, Zhang F, Wei K, et al. Fast, sensitive and accurate integration of single-cell data with Harmony. Nat Methods. 2019;16(12):1289-96.

8. Hu C, Li T, Xu Y, Zhang X, Li F, Bai J, et al. CellMarker 2.0: an updated database of manually curated cell markers in human/mouse and web tools based on scRNA-seq data. Nucleic Acids Res. 2023;51(D1):D870-D6.
