# Supplementary material for: BRD4 modulates antimicrobial defense via non-canonical NRF2 activation in macrophages to confer protection against sepsis
Source: PLoS Pathog. 2026 Apr 30;22(4):e1014192. doi: 10.1371/journal.ppat.1014192 (PMC13155688; doi:10.1371/journal.ppat.1014192)
Supplement: Attachment — Submitted filename: rebuttal letter _02_16_26_Response to Reviewers.doc [file ppat.1014192.s019.doc]

Dear editor:

Please find enclosed a revised version of our manuscript entitled “BRD4 modulates antimicrobial defense via non-canonical NRF2 activation in macrophages to confer protection against sepsis”. We appreciated the comments suggested by reviewers. We have performed new experiments and analyses that directly address each of the requests made by the reviewers. We believe the resubmitted paper is much stronger and thank the reviewers for their insightful suggestions and criticisms.

We provide the following point-by-point response to concerns raised by each of the reviewers:

### **Reviewer 1:**

1) *“The causal relationship between BRD4 and NRF2 activation needs to be demonstrated more rigorously. The authors conclude that BRD4 stabilizes NRF2, thereby inducing MARCO/MSR1 expression, but they have not performed epistasis analyses in vivo (e.g., double knockout Brd4^f/f; LysM-Cre × Nrf2^f/f, or NRF2 overexpression rescue). To confirm that the proposed BRD4→NRF2 signaling axis is causal, additional experiments are necessary. The authors should test whether the rescue effect of SFN depends on NRF2—using either pharmacologic inhibition (ML385) in vitro or myeloid-specific Nrf2 deletion in vivo. These experiments would establish the directionality and NRF2-dependence of BRD4’s effects.”*

Response: Thank you for your insightful comments regarding the causal relationship between BRD4 and NRF2 activation.

In response to your comment about performing epistasis analyses, we conducted a study using myeloid-specific NRF2 reconstitution in *Brd4* CKO mice through the AAV9-Lyz2 system, followed by CLP to induce sepsis. Our results reveal that *Brd4* CKO mice reconstituted with NRF2 exhibited significantly reduced plasma levels of inflammatory cytokines, alleviated organ damage in the liver and lungs, and decreased bacterial burden in the peritoneum, blood, and multiple organs compared to those without NRF2 reconstitution. These findings suggest that the increased susceptibility of *Brd4* CKO mice to CLP-induced sepsis is indeed associated with NRF2 deficiency (see new Fig.8).

To further assess whether the protective effects of SFN in *Brd4*-CKO septic mice are mediated through NRF2 activation, we administered ML385, a selective NRF2 inhibitor, to SFN-treated *Brd4*-CKO mice subjected to CLP. We found that ML385 treatment reversed the SFN-mediated reduction in plasma inflammatory cytokine levels, attenuated organ injury, and increased bacterial clearance, thereby demonstrating that SFN specifically acts through NRF2 in this context. Additionally, at the cellular level, ML385 dose-dependently reversed the effects of SFN on NRF2, MARCO, MSR1 protein expression, and antibacterial activity in macrophages, further confirming the specificity of SFN for NRF2 (see new Supplementary Fig. 7 and Supplementary Figs. 6A & C).

These results have been included in the revised manuscript, providing robust evidence for the BRD4/NRF2 signaling axis and its implications in sepsis.

2) *“The direct interaction between BRD4 and NRF2 should be more conclusively demonstrated. The current co-immunoprecipitation data rely largely on overexpression in 293T cells. Endogenous BRD4–NRF2 binding should be confirmed in primary macrophages using co-IP or proximity ligation assays (PLA). To* *support the proposed KEAP1 competition mechanism, in vitro competition assays with recombinant proteins (pull-down or SPR) would be highly informative. In addition,* *genome-wide analyses (ChIP-seq or CUT&RUN) for NRF2 and BRD4 co-occupancy would strengthen the evidence for cooperative transcriptional regulation.”*

Response: We would like to thank the reviewer for the constructive comments. In response, we have conducted additional experiments that strengthen our findings:

1) In addition to performing co-IP assays in overexpressing 293T cells, we also conducted co-IP experiments using BMDMs infected with *E. coli* or *S. aureus*. These experiments clearly demonstrate a significant endogenous interaction between BRD4 and NRF2 (see new Fig. 5D & E).

2) We also employed the APEX2 proximity labeling technique, which revealed that BRD4 and NRF2 are primarily co-localized in the nucleus, supporting their functional interaction in a physiological context (see new Supplementary Fig. 5 C).

3) To further validate the proposed KEAP1 competition mechanism, we performed *in vitro* competition assays using recombinant Flag-NRF2 and Myc-KEAP1 proteins in combination with GST-tagged BRD4 deletion mutant (BRD4-PID), which contains the NRF2-interacting region. Although we could not obtain full-length GST-BRD4, GST-BRD4-PID served as an effective substitute. Our results indicate that increasing concentrations of BRD4-PID lead to a decrease in the interaction between NRF2 and KEAP1, providing evidence for competitive binding (see new Fig. 7E).

4) For genome-wide analyses of NRF2 and BRD4 co-occupancy, we utilized publicly available ChIP-seq data. Due to the lack of anti-NRF2 ChIP-seq data for LPS-treated BMDMs, we analyzed anti-NRF2 ChIP-seq data from LPS + IFNγ-treated KEAP1-deficient BMDMs as a suitable alternative. Our analysis identified 2,835 genes co-occupied by NRF2 and BRD4, with Gene Ontology (GO) enrichment highlighting pathways related to immune response activation, regulation of inflammatory response, regulation of innate immune response, and responses to bacterial molecules. KEGG pathway analysis revealed significant associations with pathways such as the NOD-like receptor signaling pathway, efferocytosis, phagosome, and lysosome, all of which align with previously reported functions of NRF2 and BRD4. This genome-wide co-occupancy analysis provides robust bioinformatic support for our model, revealing a shared transcriptional program orchestrated by BRD4 and NRF2 that is closely linked to innate immune effector functions in macrophages.

It is worth noting that while we identified MACRO among the co-occupied genes, MSR1 was not present. This discrepancy likely reflects context-dependent regulatory mechanisms that merit further exploration.

We have included these new findings and discussions in the revised manuscript to address the reviewer's concerns comprehensively.

3) *“**The SFN rescue experiments are compelling but need to rule out off-target effects. Because SFN affects multiple pathways, the authors should clarify whether its protective effects are strictly NRF2-dependent. Combining SFN with NRF2 inhibition, and testing different doses or treatment timing, would improve translational relevance and mechanistic specificity. Although SFN improved survival in both WT and Brd4-CKO mice, this does not by itself prove NRF2 exclusivity—clarifying this would strengthen the conclusion.”*

Response: To address your concerns, we have conducted additional experiments that further investigate the specificity of SFN in relation to NRF2. As previously mentioned, we administered ML385, a selective NRF2 inhibitor, to SFN-treated *Brd4*-CKO mice subjected to CLP. Our findings indicate that ML385 effectively reversed the protective effects of SFN, confirming that SFN's protective effects are primarily mediated through NRF2 activation (see new Supplementary Fig. 7).

Additionally, we have incorporated dose-response *in vitro* studies to evaluate the impact of varying concentrations of ML385 on the reversal of SFN's effects. These findings consistently support the conclusion that the protective effects of SFN are indeed NRF2-dependent, reinforcing our insights into the BRD4/NRF2 signaling axis (see new Supplementary Figs. 6A & C).

We will ensure that these clarifications and results are clearly articulated in the revised manuscript to strengthen our conclusions and enhance the translational relevance of our findings.

4) *“**Concerning the LysM-Cre model, the manuscript should acknowledge its known limitations. LysM-Cre drives recombination in both macrophages and neutrophils; thus, the phenotype might partly reflect altered neutrophil function. Assessing neutrophil phagocytosis, bactericidal activity, or NETosis—or reproducing key experiments using a macrophage-specific driver (e.g., Cx3cr1-CreER)—would help to exclude this confounding factor.”*

Response: We sincerely appreciate the valuable suggestion regarding clarifying the cell-type specific contributions of the LysM-Cre model. In response to this recommendation, we systematically assessed the antimicrobial functions of neutrophils in Brd4 CKO mice. Our findings reveal that BRD4 deficiency significantly impairs both phagocytic and bactericidal activities in neutrophils (see new Supplementary Fig. 9). These results indicate that BRD4 is not exclusively critical for macrophages but serves as a vital regulatory hub for the antimicrobial functions of myeloid innate immune cells.

The protective role of BRD4 in the CLP model arises from its dual support of both macrophages and neutrophils, which explains the pronounced susceptibility observed in Brd4 CKO mice, a reflection of a cumulative immune deficiency at the multicellular level. Importantly, while the gene knockout experiments underscore the necessity of BRD4 for neutrophil bactericidal function, we noted that the baseline mRNA levels of Brd4 in neutrophils from septic patients do not correlate significantly with systemic inflammatory markers such as CRP and PCT. This suggests that BRD4 may regulate the rapid antimicrobial responses of neutrophils through post-translational modifications or dynamic activity rather than solely through baseline expression levels. We have incorporated this critical analysis into the revised manuscript in the Discussion section (lines 539–550).

Thank you again for your insightful feedback, which has greatly enhanced the clarity and depth of our work.

5) *“The human data are of high translational value but require better statistical control. Multivariate analyses adjusting for age, sex, infection source, comorbidities, and treatment variables should be presented to confirm the independent association between BRD4 (and NRF2) expression and clinical severity. Receiver operating characteristic (ROC) analysis for prognostic performance would also add rigor. Furthermore, sampling timing and disease stage heterogeneity should be clarified, and longitudinal analysis of expression dynamics would be valuable.”*

Response：We sincerely appreciate your constructive suggestions, which have significantly enhanced the statistical rigor of our human data analysis.

In response to your comments regarding multivariable analysis, we have adjusted for age and sex in our analyses. Although information on infection source and comorbidities was not available in the clinical datasets we utilized, our adjustments confirmed that the downregulation of *BRD4* transcripts in septic patients remains robust. Additionally, we conducted a receiver operating characteristic (ROC) analysis, which validated the strong discriminative performance of BRD4 in distinguishing septic patients from healthy controls. We also performed a longitudinal analysis using data from Days 1, 3, and 5, which revealed that BRD4 expression reaches its nadir at disease onset and progressively increases throughout the clinical course. This information is included in the revised manuscript (lines 140-146).

Regarding NRF2, after adjusting for age and sex, we found no statistically significant difference in NRF2 expression between septic non-survivors and survivors; thus, we have removed this data from the revised manuscript. However, new findings presented in Figures 9H-J demonstrate that NRF2 expression in monocytes from PBMCs exhibits a significant inverse correlation with SOFA scores, suggesting that lower NRF2 expression may be associated with more severe sepsis. Moreover, NRF2 expression positively correlates with MARCO expression in the whole blood of septic patients. Notably, MARCO expression was significantly lower in non-survivors compared to survivors, remaining robust after adjusting for age and sex. These findings indicate that NRF2 may represent a potential therapeutic target for sepsis in humans.

Thank you once again for your valuable feedback, which has led to important improvements in our manuscript.

6) *“Mechanistically, the authors could explore how BRD4 inhibits NRF2 ubiquitination beyond KEAP1 competition—whether it recruits deubiquitinases or influences* *Cullin3 E3 ligase activity.”*

Response: We thank the Reviewer for raising this insightful question regarding the precise mechanism by which BRD4 stabilizes NRF2. We conducted new experiments to further investigate this mechanism and would like to share our findings:

1) BRD4 knockout did not affect the interaction between NRF2 and known deubiquitinases such as USP7, USP11, and DUB3, which further supports that BRD4 does not recruit these deubiquitinases to NRF2 (see new Supplementary Figs. 12B-D).

2) Additionally, BRD4 deletion does not impact the formation of the KEAP1-CUL3 complex or the neddylation status of CUL3, indicating that BRD4 does not influence Cullin3 E3 ligase activity in this context (see new Supplementary Figs. 12A).

These findings suggest that BRD4's inhibitory effect on NRF2 ubiquitination is not mediated through the recruitment of deubiquitinases, or modulation of Cullin3 activity. We have included this detailed discussion and supplementary data in the revised manuscript to clarify the mechanistic insights regarding BRD4's role in NRF2 regulation.

7) *“In the discussion, the authors should elaborate on the dual nature of BRD4—as both an enhancer of inflammatory gene expression and a protector of antimicrobial function. Presenting a phase-specific therapeutic framework (inhibiting BRD4 in early hyperinflammation, activating it during the immunosuppressive phase) would increase clinical relevance. Additionally, potential risks of sustained NRF2 activation (tumor promotion, metabolic effects) should be briefly mentioned.”*

Response: We sincerely thank the reviewer for this excellent and clinically insightful suggestion. We have elaborated on the dual nature of BRD4 in our discussion, highlighting its role as both an enhancer of inflammatory gene expression and a protector of antimicrobial function. This addition can be found in the revised manuscript (lines 532-538).

Furthermore, we have introduced a phase-specific therapeutic framework, proposing that inhibiting BRD4 during the early hyperinflammatory phase could mitigate excessive inflammation, while strategically activating it during the subsequent immunosuppressive phase may enhance antimicrobial functions. This framework is detailed on lines 649-655 of the revised manuscript.

Additionally, we have addressed the potential risks associated with sustained NRF2 activation, including tumor promotion and metabolic effects. This discussion is included on lines 637-642, providing a balanced perspective on the therapeutic targeting of NRF2.

**Reviewer 2:**

1) *“Figure 3A, it is not clear the bacterial burden was measured under anaerobic or aerobic culture condition? If the colony-formed Gram-negative and -positive bacteria could be isolated and identified, would they be more relevant species than E. coli and S. aureus to assess the myeloid-specific impact of BRD4 in bacterial clearance and sepsis, as in Figure 3?”*

Response: Thank you for your valuable feedback regarding our manuscript. We apologize for the lack of clarity in the original description. Bacterial burden was quantified under aerobic conditions (ambient air, ~21% O₂), as reported in the literature (PMID: 30995475). This information has now been explicitly added to the revised Methods section.

Recognizing that CLP polymicrobial sepsis involves both aerobic and anaerobic bacteria, we performed additional experiments under microaerobic conditions (10% CO₂, 5% O₂, 85% N₂). Consistent with the aerobic results, *Brd4* CKO mice exhibited significantly higher bacterial loads than WT controls under microaerobic culture. Notably, microaerobic conditions revealed additional small colonies, likely representing facultative or obligate anaerobes.

We agree that identifying the actual bacterial species in the CLP model would strengthen the study. To address this, we performed 16S rDNA sequencing on blood from CLP-induced WT mice. The most abundant species was s_Unclassified_g_*Escherichia*, consistent with the predominance of *E. coli* in CLP-derived sepsis. Notably, we did not detect *Staphylococcus* in our sequencing dataset. This may reflect limited sequencing depth or dynamic shifts in the microbiota during CLP sepsis, as *Staphylococcus* has been reported in mouse cecal contents (PMID: 38242132).

For in vitro mechanistic studies, we selected *E. coli* and *S. aureus* as prototypes of Gram-negative and Gram-positive bacteria, respectively, based on prior established literature (PMID: 28922428). Moreover, *S. aureus* remains a clinically relevant pathogen in human sepsis. We believe this approach provides a reasonable and widely accepted framework for dissecting myeloid-specific BRD4 function. We have now added a discussion of these points in the revised manuscript (lines 495–505).

*de Tymowski C, Heming N, Correia MDT, Abbad L, Chavarot N, Le Stang MB, et al. CD89 Is a Potent Innate Receptor for Bacteria and Mediates Host Protection from Sepsis. Cell Rep. 2019;27(3):762-75 e5.* (PMID: 30995475)

*Xu D, Zhou S, Liu Y, Scott AL, Yang J, Wan F. Complement in breast milk modifies offspring gut microbiota to promote infant health. Cell. 2024;187(3):750-63 e20.* (PMID: 38242132)

*Jin L, Batra S, Jeyaseelan S. Diminished neutrophil extracellular trap (NET) formation is a novel innate immune deficiency induced by acute ethanol exposure in polymicrobial sepsis, which can be rescued by CXCL1. PLoS Pathog. 2017;13(9):e1006637.* (PMID: 28922428)

2) *“Myeloid-specific Brd4 knockout mice was previously shown resistant to LPS-induced sepsis, while the current study demonstrated that Brd4 conditional knockout mice were more susceptible to CLP-induced polymicrobial sepsis (ref 21). Such phenotype difference should be discussed in depth, in particular, the identification of the recruitment of Brd4 to the promoters of Marco and Msr1 was based on the LPS treatment data (Supplementary Figure 5 and Figure 5A).”*

Response: Thank you for your insightful comments regarding the contrasting phenotypes of *Brd4* -CKO mice in LPS-induced endotoxemia versus CLP-induced polymicrobial sepsis. In the revised manuscript, I have addressed this issue in the discussion section (lines 525-538).

I elaborated on how these differing phenotypes reflect the dual roles of BRD4 in modulating inflammatory and antimicrobial responses. Specifically, BRD4 enhances pro-inflammatory cytokine production, and its deletion mitigates the lethal cytokine storm during LPS exposure, thereby providing protection. Conversely, effective defense against live bacterial infections necessitates robust bacterial clearance. Our findings indicate that BRD4 deficiency hampers phagocytosis through the downregulation of scavenger receptors MARCO and MSR1, which are induced by LPS and regulated by BRD4 at the promoter level.

While our analysis of public ChIP-seq data was based on LPS stimulation, we confirmed BRD4 recruitment to these promoters during bacterial infection (Figs. 5B & C). Importantly, both MARCO and MSR1 knockout mice demonstrate resistance to LPS-induced pathology, highlighting the context-dependent nature of these outcomes. Thus, BRD4 plays a critical role in balancing inflammation and antimicrobial defense, with the overall impact on host survival being influenced by the specific type of challenge encountered.

Thank you once again for your valuable feedback, which has strengthened our discussion.

3) *“What were the transcription levels of Brd4 in Figure 1 J and K? It would be necessary to ascertain whether the decreased levels of BRD4 in later time points could result from the protein degradation.”*

Response: Thank you for your insightful comments regarding the transcription levels of *Brd4*. The mRNA levels of *Brd4* exhibit a similar trend to the protein levels, initially increasing during bacterial infection before declining (see new Supplementary Fig. 1D). This suggests that the reduction in BRD4 protein levels during later time points is likely related to transcriptional regulation. However, we acknowledge that the decrease in BRD4 protein levels could also be influenced by protein degradation processes. We plan to explore this aspect further in our future research. We have included these details in the Discussion section of the revised manuscript (lines 487–491) for clarity.

We believe we have responded to each of the concerns raised by the reviewers in the revised manuscript, and we have included a number of new experiments as requested. We hope that our manuscript is now suitable for publication in PLOS Pathogens and look forward to your editorial decision.

Sincerely,

Xiangming Hu

Professor of School of Basic Medical Sciences

Fujian Medical University
