# Supplementary material for: BRD4 modulates antimicrobial defense via non-canonical NRF2 activation in macrophages to confer protection against sepsis
Source: PLoS Pathog. 2026 Apr 30;22(4):e1014192. doi: 10.1371/journal.ppat.1014192 (PMC13155688; doi:10.1371/journal.ppat.1014192)
Supplement: Attachment — Submitted filename: rebuttal letter _04_03_26_Response to Reviewers.doc [file ppat.1014192.s020.doc]

Dear editor:

We would like to express our sincere gratitude to you and the reviewers for your valuable comments and feedback on our manuscript titled “BRD4 modulates antimicrobial defense via non-canonical NRF2 activation in macrophages to confer protection against sepsis”. We have carefully addressed all the comments and revised the manuscript accordingly. Below is a summary of our responses to each reviewer’s comments:

### **Reviewer #1:**

*“The authors have addressed the concerns and critiques in a satisfying manner. The revised version of the manuscript with newly added experimental data and extensive revisions is suitable for publication.”*

We are grateful to Reviewer #1 for the positive assessment, indicating that all concerns have been satisfactorily addressed.

**Reviewer #2:**

1. *“The ChIP-seq analysis supporting BRD4–NRF2 co-occupancy relies on publicly available datasets. While this analysis provides useful bioinformatic support, the authors may clarify this limitation in the manuscript and discuss potential context-dependent differences between experimental systems.”*

We agree that using publicly available ChIP-seq datasets has inherent limitations. We have addressed this comment by adding the following text to the Discussion section (lines 582–600 of the revised manuscript):

“To gain a genome-wide perspective on cooperative transcriptional regulation by BRD4 and NRF2, we analyzed publicly available ChIP-seq data. We acknowledge that this bioinformatic approach has inherent limitations, including the absence of anti-NRF2 ChIP-seq data from LPS-treated BMDMs. As an alternative, we analyzed anti-NRF2 ChIP-seq data from LPS+IFNγ-treated KEAP1-deficient BMDMs, in which NRF2 is stabilized and nuclear-localized, partially mimicking the BRD4-mediated NRF2 stabilization we propose. This analysis identified 2,835 DNA regions co-occupied by BRD4 and NRF2 (Supplementary Figs. 12A & B). GO enrichment revealed pathways related to immune response activation, regulation of inflammatory response, and response to bacterial molecules (Supplementary Fig. 12C). KEGG pathway analysis showed significant associations with NOD-like receptor signaling, efferocytosis and phagosome (Supplementary Fig. 12D), consistent with known NRF2 and BRD4 functions. Thus, this analysis provides bioinformatic support for a shared transcriptional program linked to innate immune effector functions. Notably, while *Marco* was identified among co-occupied genes, *Msr1* was not. This discrepancy likely reflects context‑dependent regulatory mechanisms, possibly arising from differences in cellular stimulation conditions. Future studies using stimulation-matched ChIP-seq will be required to fully validate the direct co-regulation of specific target genes.”

2. *“In the KEAP1 competition assays, a BRD4 deletion construct (BRD4-PID) was used instead of full-length BRD4. The authors may briefly discuss whether additional domains of BRD4 could influence NRF2 binding in vivo.”*

We thank the reviewer for this important point. To address whether other domains of BRD4 influence NRF2 binding, we first examined the contribution of the bromodomains (BD1 and BD2). As shown in Figure 6I, deletion of both BD1 and BD2 did not affect the BRD4-NRF2 interaction, indicating that these domains are dispensable for binding. Furthermore, our truncation analysis (Figure 7B) revealed that BRD4 constructs lacking the C-terminal region (1–1279 and 1–1328) almost completely lost NRF2 binding, whereas the 1–1334 mutant, which retains the RKR motif, showed partially restored binding. These data demonstrate that the C-terminal ~30–40 amino acids are the primary mediator of the BRD4-NRF2 interaction. Based on this, we generated a BRD4 deletion mutant (BRD4-PID, amino acids 1302–1362, corresponding to the C-terminal 61 residues) encompassing this minimal binding region. Because GST-tagged full-length BRD4 was unavailable, we used GST-BRD4-PID as a surrogate. Importantly, BRD4-PID bound to NRF2 and recapitulated the inhibitory effect of WT BRD4 on the NRF2–KEAP1 interaction in a dose-dependent manner (new Figure 7G). Moreover, increasing amounts of GST-BRD4-PID progressively diminished recombinant NRF2 binding to KEAP1 (Figure 7H). These results, together with the bromodomain deletion data, indicate that the C-terminal region of BRD4 is necessary and sufficient for NRF2 binding and that other domains do not significantly contribute to this interaction in vivo. These results are now described in the Results section (lines 368-370 and lines 385–391 of the revised manuscript).

3. *“The discussion of the LysM-Cre model would benefit from a brief statement acknowledging that both macrophages and neutrophils contribute to the observed phenotype, as suggested by the newly added neutrophil functional assays.”*

We agree with the reviewer and have added the following statement to the Discussion section (lines 542–549 of the revised manuscript).

“*Brd4*-CKO mice were generated using LysM-Cre-mediated deletion, resulting in the absence of BRD4 in both macrophages and neutrophils. We assessed the antimicrobial functions of neutrophils in these mice and found that BRD4 deficiency significantly impairs their phagocytic and bactericidal activities (Supplementary Fig. 9). These findings indicate that BRD4 is crucial not only for macrophages but also for the antimicrobial functions of myeloid innate immune cells, including neutrophils. The protective role of BRD4 in the CLP model stems from its support of both cell types, explaining the heightened susceptibility in *Brd4*-CKO mice.”

Once again, we thank both reviewers for their thoughtful comments, which have significantly contributed to the enhancement of our manuscript.

We look forward to your favorable consideration of our revised manuscript.

Sincerely,

Xiangming Hu

Professor of School of Basic Medical Sciences

Fujian Medical University
